# Selective Knockdown of Ceramide Synthases Reveals Opposite Roles of Different Ceramide Species in Cardiac Homeostasis

**DOI:** 10.3390/metabo15090584

**Published:** 2025-08-31

**Authors:** Alexandra M. Wiley, Melissa A. Krueger, Jessica O. Becker, Matthew Karasu, Nona Sotoodehnia, Jason G. Umans, Andrew N. Hoofnagle, Sina A. Gharib, Rheem A. Totah, Rozenn N. Lemaitre

**Affiliations:** 1 Department of Medicinal Chemistry, University of Washington, Seattle, WA 98195, USA; 2 Computational Medicinal Core, Center for Lung Biology, Division of Pulmonary, Critical Care and Sleep Medicine, Department of Medicine, University of Washington, Seattle, WA 98109, USA; krueger@uw.edu (M.A.K.);; 3 Department of Laboratory Medicine and Pathology, University of Washington, Seattle, WA 98195, USA; becker78@uw.edu (J.O.B.); ahoof@uw.edu (A.N.H.); 4 Cardiovascular Health Research Unit, Department of Medicine, Division of Cardiology, University of Washington, Seattle, WA 98195, USA; nsotoo@uw.edu (N.S.); rozenl@uw.edu (R.N.L.); 5 MedStar Health Research Institute, Hyattsville, MD 21044, USA; jgu@georgetown.edu

**Keywords:** cell signaling, ceramides, lipids, sphingolipids, LC-MS, cardiovascular disease, transcriptomics

## Abstract

**Background/Objectives:** Sphingolipids are a class of lipids that play important structural and functional roles in the cell. Specific ceramide species are distinguishable through the fatty acid that is acylated to the sphingosine backbone, leading to distinct biological activities. Generally, long-chain (LC) ceramides (16:0 and 18:0) drive metabolic dysfunction resulting in the progression of different disease states, while very long-chain (VLC) ceramides (22:0 and 24:0) are thought to be either beneficial against disease progression or benign. In this study, we sought to alter the cellular composition of LC and VLC ceramides in ventricular HCMs to investigate how alterations in these lipids can affect the transcriptome of otherwise healthy HCMs. **Methods:** Here, we used specific siRNA to knockdown the ceramide synthases responsible for the production of LC and VLC ceramides in ventricular HCMs and investigated the changes in the transcriptome of HCMs with *CERS2* or *CERS5/6* silenced compared to control conditions. **Results:** Knocking down *CERS2* led to an increase in cell death as well as widespread reductions in cellular VLC sphingolipids. Additionally, we demonstrated that VLC sphingolipid species may play a protective role in maintaining cardiovascular function and that reducing these lipids may contribute to cardiac dysfunction. Similarly, knocking down CERS5 and CERS6 led to reduced LC ceramides and also resulted in profound changes in gene transcription. Interestingly, multiple genes and pathways were affected in the opposite direction when compared to the changes observed with the *CERS2* knockdown. **Conclusions:** Taken together, our results suggest pathways through which VLC ceramides may contribute to cardiac protection, and pathways where LC ceramides may promote HCM stress and the development of cardiac disease.

## 1. Introduction

Ceramides are a subclass of lipids known as sphingolipids—a group of structurally diverse lipids that share a sphingoid base backbone N-acylated to fatty acids of various chain lengths (Supplemental Figure S1A). Within the sphingolipids class, there are three distinct structural subclasses—sphingoid bases and derivatives, ceramides, and complex sphingolipids—of which ceramides represent the central essential precursor of complex sphingolipids [1]. Sphingolipids are amphipathic compounds and as such control many vital cellular functions. Structurally, sphingolipids are crucial components of the plasma membrane, contributing to more complex biological roles as bioactive signaling molecules that regulate intercellular interactions [2,3]. These bioactive molecules are central mediators in several highly complex and interconnected pathways integral to cell function, although the exact mechanisms by which they contribute to maintaining cellular homeostasis are not completely elucidated.

Sphingolipids play a pivotal role in homeostasis and any disruption or dysregulation in sphingolipid utilization and metabolism may result in pathophysiological conditions [4]. As ceramides are crucial intermediates in the biosynthesis of complex sphingolipids, such as sphingomyelin (SM) and glycosphingolipids, understanding the role ceramides play in homeostasis is important. Notably, ceramides have previously been studied and identified as major regulators in cell death, primarily by arresting the cell cycle, altering membrane permeability, and inducing apoptosis [5,6,7]. Since ceramide accumulation in the membrane is postulated to impair membrane dynamics, ceramide concentrations in the cell are tightly regulated [3,4,8].

**Figure 1:**
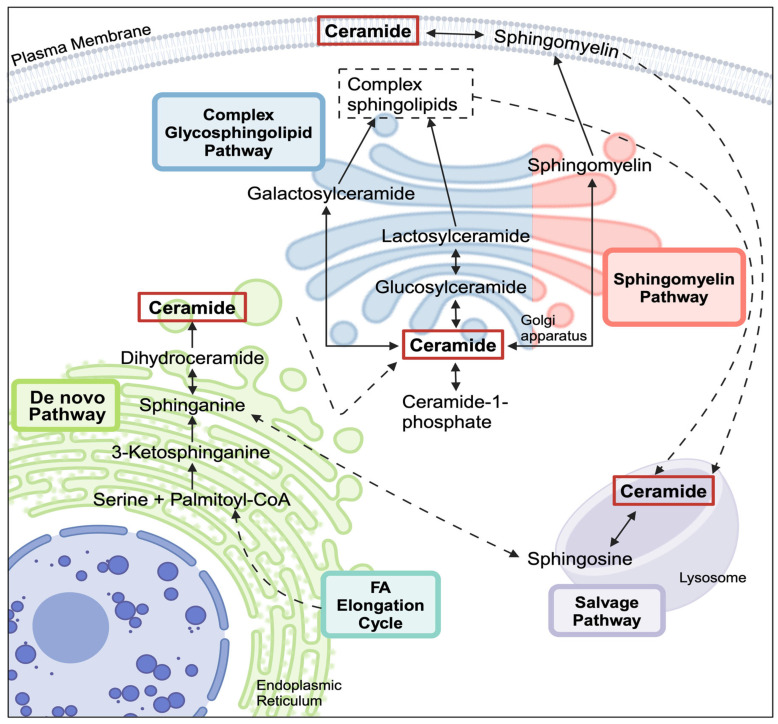
Simplified ceramide metabolic scheme. There are three main pathways through which sphingolipid metabolites are catabolized or anabolized: the *de novo* pathway, the salvage pathway, and the sphingomyelin/complex glycosphingolipid pathway. Ceramides created through these different pathways are generally thought to be spatially and functionally distinct. FA–fatty acid. Created with BioRender.com accessed on 8 August 2024.

Within the sphingolipid metabolic scheme, there are three major pathways that lead to the synthesis of ceramides—the *de novo* pathway, the sphingomyelinase pathway, and the salvage pathway (Figure 1) [9,10]. Hydrolysis of SM by sphingomyelinases in the cell membrane may contribute to circulating ceramide and SM levels, which may represent a systemic metabolic signature that reflects overall homeostasis [11,12,13,14].

Experimental evidence suggests that ceramide species with specific saturated fatty acids have distinct biological activities [15]. Although we have referred to ceramides thus far as a group, there are over 50 distinct molecular ceramide species, distinguished by their unique acyl chain length, as well as the number and location of hydroxyl groups and desaturations in the sphingoid base [3]. Additionally, at least 26 different enzymes have ceramides as either a substrate or a product, and hence have the potential to regulate ceramide levels as well as alter their concentrations and metabolites throughout the cell or body in response to stimuli [3]. Ceramide synthases (CerS) are a group of enzymes that reside in the endoplasmic reticulum and are important gatekeepers of ceramide levels. There are six mammalian ceramide synthases (CerS1-6), with each CerS displaying substrate preferences for specific fatty acid chain lengths, resulting in the formation of different N-acyl length ceramides (Supplemental Figure S1B) [16,17,18,19,20,21].

In general, long-chain (LC) ceramides 16:0 and 18:0 are thought to drive metabolic dysfunction, and high plasma concentrations have been associated with the development or progression of different disease states, including diabetes, heart failure, and Alzheimer’s disease, while very long-chain (VLC) ceramides including 22:0 and 24:0 have been suggested to be either beneficial or benign [10,21,22,23,24], depending on cell type. A global *Cers5* knockout mouse demonstrated decreased tissue 16:0 ceramide levels and resulted in reduced insulin insensitivity and glucose intolerance when challenged with a high-fat diet [25]. Similarly, knocking out *Cers6* (also responsible for the production of 16:0 ceramide) was protective against glucose intolerance in mice fed a high-fat diet [26]. Additionally, Lee et al. reported lower concentrations of 20:0, 24:0, and 24:1 ceramides and increased levels of apoptosis in mice with a cardiomyocyte specific *Sptl2* (rate-limiting step in *de novo* ceramide synthesis) knockout [27]. Thus far there has been little in vitro and in vivo work completed to aid in better understanding the physiological roles the specific ceramide species play in heart failure or other types of cardiovascular disease (CVD).

Previously, we reported that specific saturated fatty acids on plasma ceramides and SM species are differentially associated with risk of incident heart failure in the Cardiovascular Health Study, a study containing a large cohort of older adults with cardiovascular disease [28]. We discovered that 16:0 ceramide and SM were associated with an increased risk of incident heart failure, while 22:0 ceramide and 20:0, 22:0, and 24:0 SM were associated with a decreased risk of heart failure, independent of other risk factors [28]. To determine possible mechanisms underlying the associations between ceramide and SM species with incident heart failure, we hypothesized that modulating levels of 16:0, 22:0, and 24:0 ceramide in HCMs would modify biological processes relevant to heart failure. Here, we present reproducible and reliable conditions for the targeted silencing of the ceramide synthases (*CERS2* and *CERS5/6*) responsible for producing 16:0, 22:0, and 24:0 ceramide in HCMs. We further investigated how reducing these ceramide species altered overall cell survival and analyzed the whole genome transcriptional response in order to identify specific perturbed pathways that could contribute to changes in cardiac homeostasis.

## 2. Materials and Methods

### 2.1. Materials

Immortalized ventricular human cardiomyocytes (HCMs) and all their applicable materials including PriGrow I media, penicillin/strep, and extracellular matrix were purchased through Applied Biological Materials (Bellingham, WA, USA). *CERS2* siRNA duplex (#SR323951) was purchased from Origene (Rockville, MD, USA) and the universal scrambled negative control siRNA duplex that came with this product was used as the control. Silencer siRNA for *CERS5* (#AM16708, siRNA ID #131807), *CERS6* (#AM16708, siRNA ID #149485), and the control used for this experimental group, Silencer Negative Control #5 siRNA (#AM4642), were purchased from Invitrogen (Waltham, MA, USA). A small-volume protein assay was obtained from Bio-Rad Laboratories to normalize tonormalize cellular protein content prior to LC-MS/MS analysis (Hercules, CA, USA). Tissue culture treated plates, Lipofectamine 3000 Reagent, dimethyl sulfoxide (DMSO), Opti-MEM media, phosphate buffered saline (PBS), cell scrapers, PrestoBlue Viability Reagent, RNA isolation kits, and High-Capacity RNA-to-cDNA kits were all obtained from Thermo Fisher Scientific (Waltham, MA, USA). Additionally, all RT-qPCR materials were purchased from Thermo Fisher Scientific’s TaqMan Line: TaqMan Fast Advanced Master Mix for qPCR and TaqMan human primers: GUSB (Assay ID: Hs00939627_m1), *CERS2* (Assay ID: Hs00371958_g1), CERS5 (Assay ID: Hs00908759_m1), and CERS6 (Assay ID: Hs00826756_m1). Methyl tert-butyl ether, methanol, isopropanol, and LC-MS/MS grade water were from Fisher Scientific. A stable isotope-labeled internal standard mixture containing 10 sphingolipids (#LM6002) was purchased from Avanti Polar Lipids (Alabaster, AL, USA).

### 2.2. Cell Culture

Immortalized ventricular human HCMs (#T0519) were maintained with PriGrow I medium (#TM001) supplemented with 10% fetal bovine serum (FBS) and 0.1% Penicillin/Streptomycin Solution (#G255). Cells were cultured in a humidified incubator at 37 °C with 5% CO_2_. All plates were coated with Extracellular Matrix (#G422) according to the manufacturer’s protocol.

### 2.3. Silencing CERS2 and CERS5/6

Gene silencing was achieved using a reverse transfection protocol in a 6-well plate. Cells (3.5 × 10^5^) were plated with 10 nM of each respective siRNA and 5.9 µL Lipofectamine 3000 (#L3000001) per well. The lipofectamine and siRNA were mixed with Opti-MEM media (#31985070) and allowed to sit at room temperature for 20 min before addition to the cells. The transfection mixture was combined with cells suspended in complete media and a total volume of 2.5 mL was added to each well, with the transfection mixture accounting for 10% of the total volume. After 24 h cells either underwent a media change (for mass spectrometry analysis) or a 48 h vehicle treatment was initiated in serum-free media (maximum 0.025% DMSO).

Sphingolipid quantification was achieved by high-performance liquid chromatography-tandem mass spectrometry.

Following transfection for 48 h, cells were washed twice with 600 µL ice cold PBS (#10010031), harvested by scraping in ice cold PBS, and pelleted by centrifugation at 300× *g* for 5 min. The supernatant was then aspirated and the cell pellets were stored at −80 °C until further analysis. Each experiment was performed in triplicate (*n* = 3).

To increase rigor, experiments were repeated three months later (*n* = 6 for each experimental condition). Once cell pellet collection was complete, the samples were thawed on ice and resuspended in PBS. The cells were then lysed by placing them in an iced sonication bath for 30 min. Each sample was normalized to 1 mg/mL following measurement with a small volume protein assay (#500012).Sphingolipids in each cell lysate were quantified using solvent extraction with a methyl tert-butyl ether, methanol, isopropanol mixture, and measured via liquid chromatography-tandem mass spectrometry, as previously described in detail [29]. Statistical significance was determined through multiple unpaired *t*-tests.

### 2.4. Total RNA Isolation and RT-qPCR

Following the 24 h siRNA incubation and the completion of the 48 h vehicle treatment, cells were rinsed once with ice cold PBS and lysed with TRI Reagent. The cells then underwent total RNA isolation utilizing the MagMax-96 for Microarrays Total RNA Isolation Kit (#AM1839) following the spin procedure. After RNA isolation, RNA was converted to cDNA by reverse transcriptase using the High-Capacity RNA-to-cDNA kit (#4387406). RT-qPCR was then conducted utilizing the Taqman Gene Expression Assay reagents (Thermo Fisher Scientific, FAM dye). Gene expression was normalized to the housekeeping gene, GUSB, and data was analyzed following the comparative delta-Ct method. Unpaired two-tailed *t*-tests were used to determine statistical significance between samples.

### 2.5. Cell Viability

Cell viability was measured in HCMs that underwent a scaled-down transfection, with respect to surface area, in a 24-well plate. Every 24 h, for a total of 72 h post transfection, wells underwent a fresh serum-free media exchange with the vehicle control (maximum 0.025% DMSO). At the 72 h mark, HCM viability was measured utilizing the PrestoBlue HS Cell Viability Reagent (#P50201) according to the manufacturer’s protocol. HCMs were incubated in the presence of the resazurin-based presto blue reagent for 2 h prior to reading the absorbance using the Tecan Spark V3.0. Statistical significance was determined using unpaired two-tailed *t*-tests.

### 2.6. Library Preparation and mRNA-Sequencing

After total RNA isolation, samples were sent to Novogene Corporation Inc. (Sacramento, CA, USA) for mRNA-sequencing. Samples were first subjected to a quality control check and library preparation, where samples underwent a polyA capture to enrich mRNA and remove rRNA, then the mRNA was converted into cDNA. The libraries then underwent a second quality control before sequencing with Illumina PE150 technology. Sequencing data was checked for quality a final time before undergoing bioinformatic analysis. The sequence of each sample was aligned to the human reference genome GRCh38 and all samples were confirmed to map to over 90% of the reference genome. One of the scramble controls for the *CERS2* KD did not meet this criterion, and hence was excluded from further analysis.

### 2.7. Bioinformatics and Pathway Analysis

#### 2.7.1. Gene Expression Analysis

Once the sequence was mapped to the reference genome, the raw counts were filtered by removing any genes that did not have a maximum value of at least 10 across all samples. Differentially expressed genes were then identified between the various treatment groups and controls using the DESeq2 package in R and significance was assigned by using a Benjamini–Hochberg adjusted *p*-value < 0.01 [30].

#### 2.7.2. Gene Set Enrichment Analysis (GSEA)

To identify biological pathways altered by each *CERS* KD condition, we applied GSEA [31,32] using two Molecular Signature Database categories: Hallmark and Canonical Pathways [33,34]. The pre-ranked genes were ordered based on DESeq2’s test statistic [30] and a false discovery rate (FDR) < 0.05 threshold was implemented to designate significant gene set enrichment.

Additionally, the list of genes compiled within the gene set entitled ‘GOBP sphingolipid metabolic process’ was utilized for sphingolipid differentially expressed gene (DEG) investigation. These genes were separated into 6 different groups based on their role within sphingolipid metabolism: fatty acid (FA) elongation cycle, *de novo* pathway, salvage pathway, sphingomyelin pathway, complex sphingolipid pathway, or, if the gene did not fit into any of these sectors of the metabolic scheme, it was placed into an “other” sphingolipid pathway group. Following the completion of the GSEA pathway analyses, enrichment maps were created utilizing Cytoscape 3.10.2 with all identified pathways FDR < 0.05 [35] for both *CERS2* (Supplemental Figure S5) and *CERS5/6* KDs (Supplemental Figure S8). The distinct biological modules that emerged were used to label the volcano plots demonstrating pathway changes due to each KD.

## 3. Results

To confirm the expression of the different *CERS* in the HCMs in culture, we utilized RNA sequencing data (Supplemental Figure S2). Notably, *CERS2* expression was the highest, followed by *CERS5* and *CERS6* expression. Since *CERS2* is responsible for the production of the proposed protective (22:0 and 24:0) ceramides, and CerS5/CerS6 are both responsible for the generation of the suggested detrimental (16:0) ceramide, we divided the experimental groups up as such, combining the knockdowns of *CERS5/6*.

### 3.1. Ceramide Synthase 2 (CERS2) Knockdown

Silencing *CERS2* by 80% led to significant reductions in the VLC ceramide levels, as expected. Knockdown conditions were optimized to maximize differences observed in the cellular response; therefore, we aimed for an average of ~80% *CERS2* KD (Supplemental Figure S3A), which was consistent over time. While optimizing the *CERS2* KD, we observed significant cell death with 80% KD (Supplemental Figure S3B). Of note, to minimize cell death, we also optimized conditions leading to 40–50% *CERS2* KD. These conditions resulted in a lower percentage of cell death but we did not observe changes in the VLC ceramide levels following sphingolipid quantification. Therefore, despite the increase in cell death, which confines our conclusions, we chose to conduct follow up experiments with ~80% *CERS2* KD. We did take extra measures to extensively wash the cell pellet to remove all dead cells prior to LC-MS/MS quantification and RNA isolation below.

**Figure 2:**
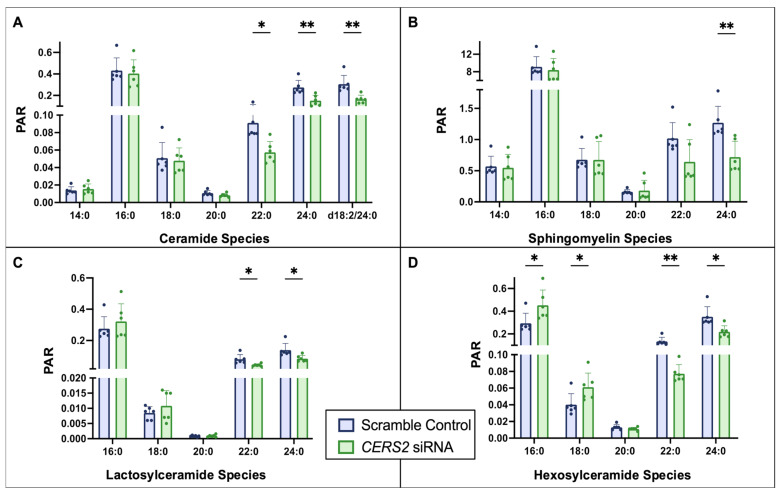
Quantification of sphingolipid species changes with 72 h *CERS2* KD according to class: (**A**) ceramide, (**B**) sphingomyelin, (**C**) lactosylceramide, and (**D**) hexosylceramide. Unless otherwise noted, all species had an 18:1 sphingosine backbone. Two sets of *n* = 3 sample collections resulting in an *n* = 6, ** *p* < 0.01, * *p* < 0.05, PAR—peak area ratio.

A closer look at the different sphingolipid species measured suggests overall reductions in 22:0 and 24:0 sphingolipids with *CERS2* KD (Figure 2). As expected, we observed significant decreases in 22:0 and 24:0 ceramide as well as other sphingolipid species with an acylated 22:0 or 24:0 FA. We also detected increased amounts of 16:0 and 18:0 hexosylceramides in *CERS2* KD compared to controls.

### 3.2. Global Transcriptomic Changes Following CERS2 Knockdown

**Figure 3:**
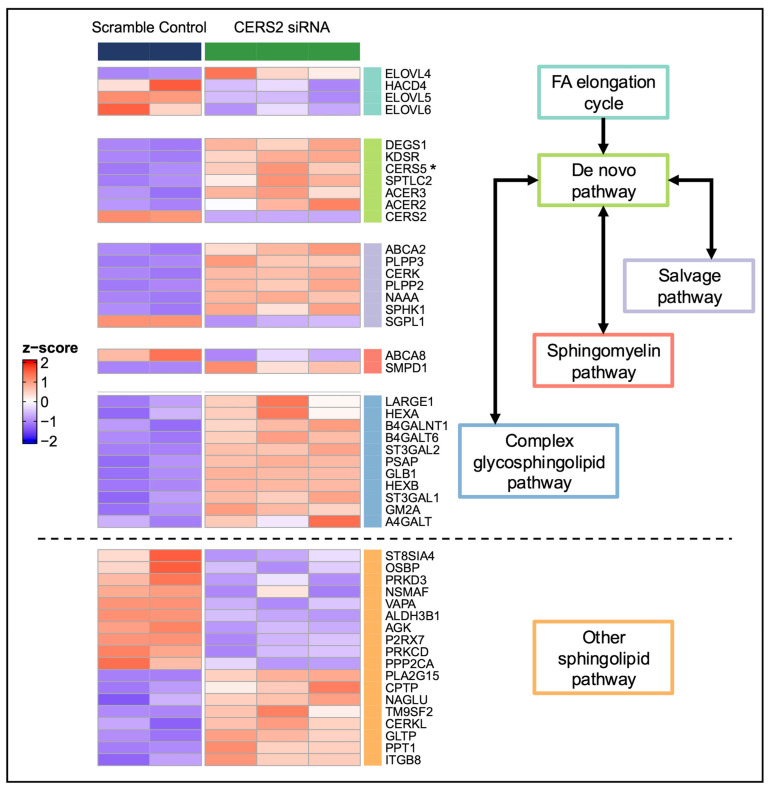
DEGs within the sphingolipid metabolic scheme identified following *CERS2* KD. Genes important to sphingolipid metabolism (adjusted *p*-value < 0.05) are highlighted due to *CERS2* KD. A positive z-score (red) indicates higher expression, while a lower z-score (blue) indicates lower expression. * Signifies potential interregulation within the *CERS*. FA—fatty acid.

To assess the global effects of *CERS2* KD in HCMs, we applied principal component analysis (PCA) to the transcriptome of HCMs with *CERS2* KD and control HCMs treated with scrambled siRNA (scramble controls). We observed clear separation between the *CERS2* KD and scramble controls, indicative of large transcriptomic changes in the HCM gene expression profiles due to KD (Supplemental Figure S4). By applying a statistical threshold (adjusted *p*-value < 0.01), we identified about 3400 DEGs. Figure 3 highlights the important DEGs (expanded to adjusted *p*-value < 0.05) that fall within the sphingolipid metabolic scheme divided up by specific functional roles, ranging from the FA elongation cycle to complex sphingolipid metabolism.

**Figure 4:**
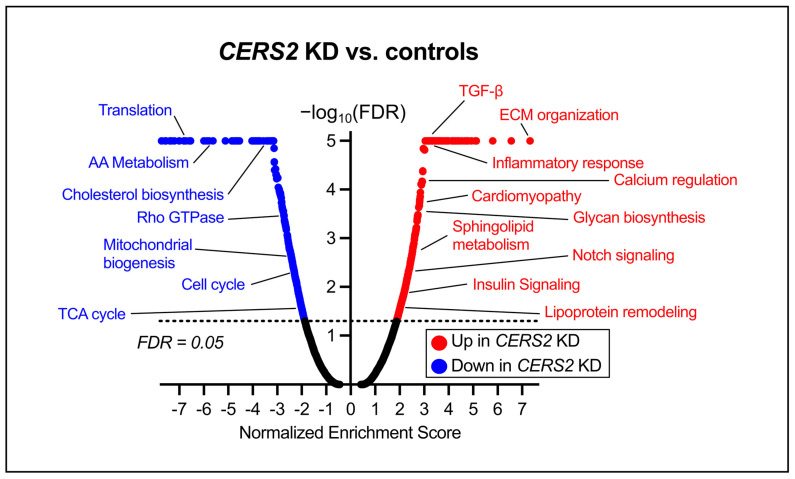
Volcano plot depiction of pathways differentially altered due to *CERS2* KD in HCMs. The labeled pathways are representative of the vast changes observed in the *CERS2* KD and further represent important pathways for heart function and cellular homeostasis. AA—amino acid, TCA—tricarboxylic acid, ECM—extracellular matrix. A full list of altered gene sets is included in Supplemental Table S1.

To further elucidate the transcriptional consequences of *CERS2* KD in HCMs, we applied GSEA to systematically identify enriched pathways among the DEGs [31,32]. We identified 420 upregulated and 328 downregulated pathways (FDR < 0.05). Figure 4 depicts a volcano plot of the diverse pathways altered due to *CERS2* KD in HCMs. Many of the differentiated pathways are important for both proper cardiac function and cellular homeostasis. In particular, following *CERS2* KD, we observed increases in pathways involved in development and remodeling, fibrosis development, and lipid dysregulation. Furthermore, we detected downregulations in genes involved in mitochondrial biogenesis, cholesterol biosynthesis, and the cell cycle.

### 3.3. Ceramide Synthase 5 and 6 (CERS5/6) Knockdown

**Figure 5:**
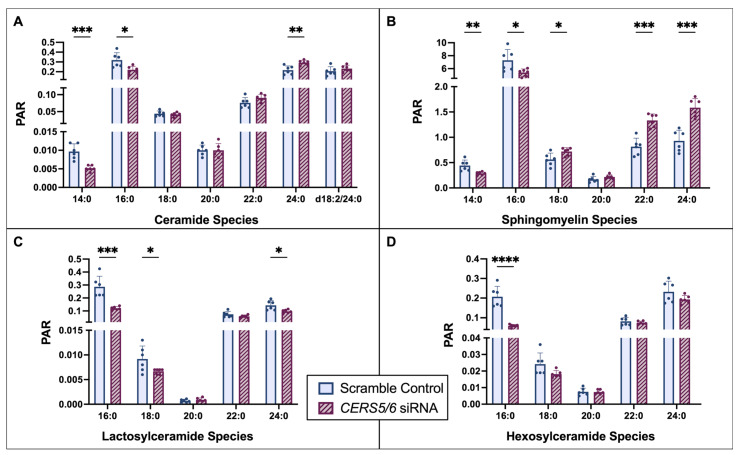
Quantification of sphingolipid species changes with 72 h *CERS5/6* KD grouped by species: (**A**) ceramide, (**B**) sphingomyelin, (**C**) lactosylceramide, and (**D**) hexosylceramide. Unless otherwise noted, all species had an 18:1 sphingosine backbone. Two sets of *n* = 3 sample collections resulting in an *n* = 6, **** *p* < 0.0001, *** *p* < 0.001, ** *p* < 0.01, * *p* < 0.05, PAR—peak area ratio.

To reduce the concentrations of cellular 16:0 ceramide, a double KD, using *CERS5* and *CERS6* siRNA, was optimized, as the two proteins encoded by these genes are responsible for the production of 16:0 ceramide and both genes are observed in HCMs. Dual KD of *CERS5* and *CERS6* at ~80% each (Supplemental Figure S6A) leads to a significant reduction in 16:0 ceramide and a 25% reduction in cell viability (Supplemental Figure S6B). With this KD, as expected, we observed significant decreases in the LC 14:0 and 16:0 ceramides when compared to controls (Figure 5). Notably, we detected overall reductions in the measured 14:0 and 16:0 sphingolipids, as well as increased VLC 24:0 ceramide and SM.

### 3.4. Global Transcriptomic Changes Following CERS5/6 Knockdown

**Figure 6:**
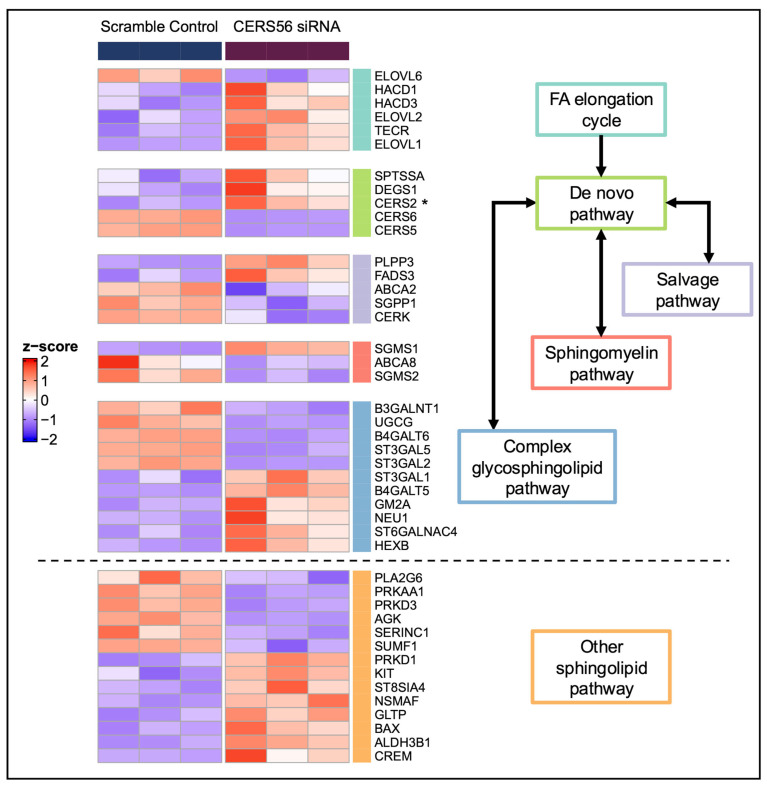
DEGs within the sphingolipid metabolic scheme identified following *CERS5/6* KD. Genes important to sphingolipid metabolism (adjusted *p*-value < 0.05) are highlighted due to *CERS5/6* KD. A positive z-score (red) indicates higher expression, while a lower z-score (blue) indicates lower expression. * Signifies potential interregulation within the *CERS*. FA—fatty acid.

We applied PCA to the entire HCM transcriptome comparing cells with *CERS5/6* KD and scramble controls and observed clear separation of the two groups (Supplemental Figure S7). Next, we identified approximately 3700 DEGs (adjusted *p*-value < 0.01). Important DEGs (expanded to adjusted *p*-value < 0.05) in sphingolipid metabolism differentiated between the *CERS5/6* KD and scramble control samples are depicted in Figure 6.

**Figure 7:**
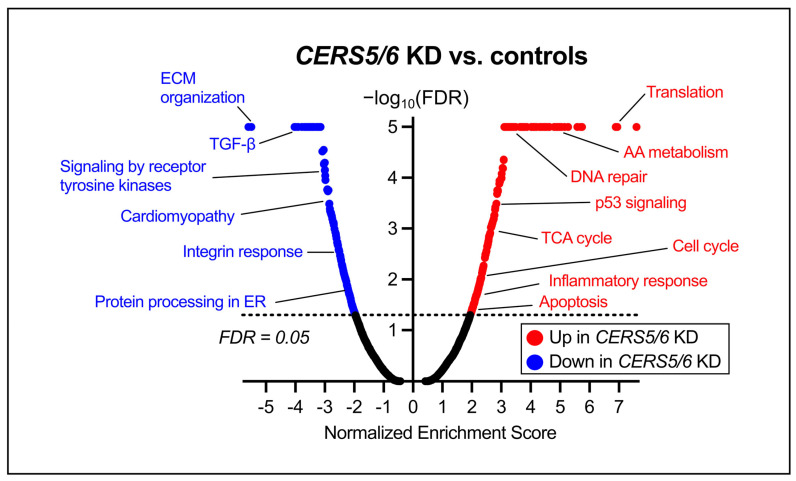
Volcano plot depiction of significantly altered pathways due to *CERS5/6* KD in HCMs with representative labeling. Many of the observed pathway changes are important for cellular homeostasis and heart function. ECM—extracellular matrix, ER—endoplasmic reticulum, AA—amino acid, TCA—tricarboxylic acid. A complete list of altered gene sets can be found in Supplemental Table S2.

GSEA uncovered many changes in pathways necessary for cellular homeostasis following *CERS5/6* KD, with 275 upregulated and 212 downregulated pathways (FDR < 0.05). A representative volcano plot is depicted in Figure 7, showcasing representative labels for many of the observed pathway changes. Interestingly, there were increases in the expression of genes and pathways involved in DNA repair, cell cycle, and p53 signaling, while pathways for development and remodeling and cardiomyopathy were downregulated with *CERS5/6* KD.

### 3.5. Discordant Transcriptional Patterns Between Different CERS Knockdowns

**Figure 8:**
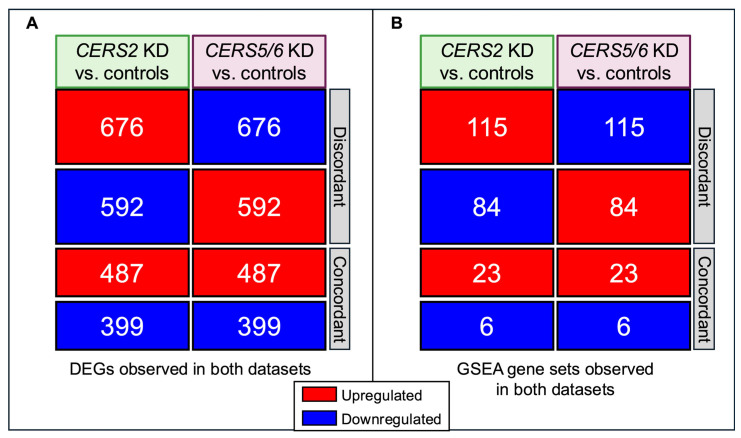
Comparison of observed changes following the KD of either *CERS2* or *CERS5/6*. Identical upregulated and downregulated (**A**) DEGs and (**B**) GSEA gene sets were independently identified with *CERS2* and *CERS5/6* KDs.

We found substantial overlap in the DEGs and pathways observed between the *CERS2* KD and *CERS5/6* KD compared to their respective controls but with inverse directionality. Of the DEGs (adjusted *p*-value < 0.01) common to both *CERS2* KD and *CERS5/6* KD, 60% were found to be discordant in directionality of their expression (Figure 8A). A similar observation was made when comparing the pathway enrichment results between *CERS2* KD and *CERS5/6* KD. Following GSEA analysis, 228 pathways were significantly changed in both the *CERS2* and the *CERS5/6* KD datasets, 87% of which had opposite enrichment patterns (Figure 8B). Taken together, these changes indicate that the different ceramide species contribute to cellular functions and homeostasis in an opposing fashion.

## 4. Discussion

In this study, we explored the consequences of altered sphingolipid homeostasis in the ventricular HCMs by silencing *CERS2* and *CERS5/6* to reduce cellular levels of 22:0 and 24:0 ceramides as well as 16:0 ceramide, respectively. The key finding in this study is that the knockdown of *CERS2* and *CERS5/6* inversely modulate critical pathways known to contribute to heart functionality. This study further supports that LC and VLC sphingolipids contribute differently to essential biological functions.

### 4.1. CERS Knockdown Leads to Intracellular Ceramide Reductions

To confirm that 80% KD led to functional changes, we quantified different sphingolipid species using LC-MS/MS following *CERS2* and *CERS5/6* KD and detected widespread changes with both conditions. With the KD of *CERS2*, as expected, we observed reductions in the potentially protective VLC ceramides, 22:0 and 24:0. Since ceramide is the central mediator of sphingolipid metabolism, it is not surprising that we observed similar reductions in the VLC complex sphingolipids: lactosylceramide (LacCer), hexosylceramide (HexCer), and SM. Interestingly, we observed a potential compensatory increase in LC HexCer following *CERS2* KD. In 2011, Mullen et al. provided evidence that the different CERS are tightly interregulated in MCF-7 cells and that *CERS2* KD resulted in potential compensatory increases in LC sphingolipids to help maintain overall cellular sphingolipid levels [36], providing further support to our findings. Overall, with *CERS2* KD, we observed the most changes in the HexCer species, which encompasses both glucosylceramide (GlcCer) and galactosylceramide (GalCer). There is experimental evidence suggesting that GlcCer levels in the heart are crucial for maintaining HCM membrane lipids, and slight alterations in GlcCer composition can contribute to reduced heart function [37]. Therefore, it is possible that the observed cell death is, in part, a consequence of altered HexCer concentrations. Of note, we did attempt to alter the KD conditions to obtain ~40% KD of *CERS2* to avoid increased cell death, but there was no observed change in any of the ceramide species analyzed by LC-MS/MS. Additionally, we attempted to shorten the KD time to 48 h, but this led to minimal changes in cell viability when compared to the 72 h KD.

With *CERS5/6* KD we observed the expected reduction in 16:0 ceramide, as well as reduction in all measured 16:0 sphingolipids: LacCer, HexCer, and SM. Interestingly, we also observed a potential compensatory increase in the VLC sphingolipids, 24:0 ceramide and SM, while 24:0 LacCer was reduced. Notably, SM concentrations were the most extensively altered sphingolipid species measured following *CERS5/6* KD. These changes in SM species may suggest an alteration in plasma membrane composition and fluidity [11,38,39].

### 4.2. Pathway Changes Within the HCMs Due to CERS Knockdown

The transcriptomic analysis with the *CERS* KDs suggests that ceramides play many vital roles within the HCMs. We observed a significant number of DEGs and importantly noted many changes in the sphingolipid metabolic scheme. In support of our findings that the VLC and LC ceramides may undergo compensatory interregulation, we observed a significant increase in *CERS5* expression with *CERS2* KD (Figure 3), while with *CERS5/6* KD, we observed a significant increase in *CERS2* expression (Figure 6).

Pathway analyses pointed to many contrasting results when comparing *CERS2* KD (Figure 4) to *CERS5/6* KD (Figure 7). With *CERS2* KD, we observed increases in HCM extracellular matrix (ECM) organization and lipid metabolism. Furthermore, we detected reduced activation of pathways that regulate mitochondrial biogenesis, cholesterol metabolism, and alter the cell cycle. Intriguingly, although these cells have not been treated with any external compounds aside from the siRNA, it appears the HCMs with silenced *CERS2* are developing an unhealthy cardiomyocyte phenotype, supported by a similar upregulation of pathways altered in human cardiomyopathy. ECM organization is indicative of cardiac remodeling [40], a hallmark for CVD pathophysiology, and dysregulations in lipid and energy metabolism may suggest the HCMs are struggling to meet the same energy requirements as healthy cardiac cells [41,42,43,44]. Taken together, these findings suggest that *CERS2* silencing has very negative impacts on HCM health.

Interestingly, we observed the opposite effect with *CERS5/6* KD, with increases in the cell cycle and energy metabolism and decreases in development and remodeling. These changes suggest that while *CERS2* KD may be detrimental to HCM health, *CERS5/6* KD may provide positive changes in HCM health and functionality. Notably, we also observed decreases in cardiovascular disease pathways, like cardiomyopathy, following *CERS5/6* KD. Taken together, these findings bolster the hypothesis that 16:0 ceramide plays a negative role in heart failure progression, while VLC ceramides 22:0 and 24:0 may play a more protective role against heart failure development [28].

A main limitation is the observed cytotoxicity with *CERS2* KD and resulting cell death. As described previously, a 40–50% KD of *CERS2* reduced cell death but did not lead to any observed changes in ceramide levels, constraining us to further explore the 80% KD with high levels of cell death. Although this is not ideal and may constrain the conclusions, we implemented wash steps to aid in sample clean up and minimize misleading signatures during transcriptomic analysis and sphingolipid quantification. It is also possible that the changes observed with the *CERS5/6* KD are, in part, due to compensatory increases in VLC sphingolipid species. Despite this limitation, the work described here contributes to understanding the important roles both LC and VLC ceramides play in maintaining cardiac homeostasis. Due to the vast number of gene alterations observed with both *CERS* KDs, we caution against the development of a CERS inhibitor as this could potentially cause toxicity with long-term treatment, similar to those observed with the mycotoxins fumonisins exposure [45,46]. For this reason, these findings are promising and suggest that the development of a *CERS2* inducer or activator could pose a viable future treatment option to combat CVD.

## 5. Conclusions

We have demonstrated that altering sphingolipid composition in HCMs leads to profound gene responses, particularly in cellular remodeling and energy metabolism. Following *CERS2* KD and extensive washing to remove dead cells, increases in ECM organization and cardiomyopathy pathways were observed as well as decreases in pathways involved in the cell cycle, while we observed opposing changes following *CERS5/6* KD despite the cell death observed with *CERS2* KD. These findings suggest that the VLC ceramides produced by *CERS2* are likely protective against CVD, while the LC ceramides produced by *CERS5/6* may have detrimental consequences to HCM health. This work lays the foundation for assessing whether altering the ceramide species through compounds directed at specific sphingolipid-producing enzymes is a viable treatment option for heart disease. Further studies focusing on modulating sphingolipid levels in HCMs and their impact on the cellular response to cardiac hypertrophy, a hallmark of heart failure progression, are needed.

## Data Availability

The RNA sequencing datasets generated and analyzed during the current study meeting MINSEQE (Minimum Information About a Next-generation Sequencing Experiment) guidelines are available in the GEO repository, [https://www.ncbi.nlm.nih.gov/geo/query/acc.cgi?acc=GSE285142], accessed 27 August 2025. Additional datasets generated and analyzed during the current study are available from the corresponding author on reasonable request.

## Supplementary Materials

The following supporting information can be downloaded at: https://www.mdpi.com/article/10.3390/metabo15090584/s1, Supplementary Table S1: List of differentially altered gene sets in *CERS2* KD HCMs (FDR < 0.05). Supplementary Table S2: List of differentially altered gene sets in *CERS5/6* KD HCMs (FDR < 0.05). Supplementary Figure S1: Acyl chain length preference of the mammalian CerS enzymes. Supplementary Figure S2: CERS gene expression in HCMs from RNA-sequencing data. Supplementary Figure S3: Optimized ~ 80% *CERS2* KD leads to significant cell death. Supplementary Figure S4: Transcriptome variation between *CERS2* KD and scramble control samples. Supplementary Figure S5: Network based visual depiction of GSEA results after *CERS2* knockdown in ventricular HCMs. Supplementary Figure S6: Optimized ~80% *CERS5/6* KD leads to small amounts of cell death. Supplementary Figure S7: Transcriptome variation between *CERS5/6* KD and scramble control samples. Supplementary Figure S8: Network based visual depiction of GSEA results after *CERS5/6* knockdown in ventricular HCMs.

## Abbreviations

The following abbreviations are used in this manuscript:

LC	long-chain
VLC	very long-chain
CerS	ceramide synthase
HCM	Ventricular human cardiomyocyte
GSEA	gene set enrichment analysis
DEG	differentially expressed gene
FDR	false discovery rate
PCA	principal component analysis
LacCer	lactosylceramide
HexCer	hexosylceramide
GlcCer	glucosylceramide
GalCer	galactosylceramide
ECM	extracellular matrix
