# Supplementary material for: Selective Knockdown of Ceramide Synthases Reveals Opposite Roles of Different Ceramide Species in Cardiac Homeostasis
Source: Metabolites. 2025 Aug 31;15(9):584. doi: 10.3390/metabo15090584 (PMC12472135; doi:10.3390/metabo15090584)
Supplement: Supplementary file 1 [file metabolites-15-00584-s001.zip › metabolites-3772496-supplementary.pdf]

Supplementary Table S1. List of differentially altered gene sets in *CERS2* KD cardiomyocytes (FDR < 0.05).

Gene Set Name	Normalized Enrichment Score (NES)	FDR
REACTOME_EXTRACELLULAR_MATRIX_ORGANIZATION	7.32	0.00E+00
HALLMARK_EPITHELIAL_MESENCHYMAL_TRANSITION	6.56	0.00E+00
HALLMARK_HYPOXIA	5.80	0.00E+00
REACTOME_REGULATION_OF_INSULIN_LIKE_GROWTH_FACTOR_IGF_TRANSPORT_AND_UPTAKE_BY_INSULIN_LIKE_GROWTH_FACTOR_BINDING_PROTEINS_IGFBPS	5.13	0.00E+00
PID_INTEGRIN1_PATHWAY	5.09	0.00E+00
REACTOME_COLLAGEN_FORMATION	4.93	0.00E+00
REACTOME_GLYCOSAMINOGLYCAN_METABOLISM	4.80	0.00E+00
KEGG_ECM_RECEPTOR_INTERACTION	4.74	0.00E+00
REACTOME_DEGRADATION_OF_THE_EXTRACELLULAR_MATRIX	4.67	0.00E+00
REACTOME_ECM_PROTEOGLYCANS	4.66	0.00E+00
REACTOME_HEMOSTASIS	4.55	0.00E+00
HALLMARK_TNFA_SIGNALING_VIA_NFKB	4.44	0.00E+00
REACTOME_CELL_SURFACE_INTERACTIONS_AT_THE_VASCULAR_WALL	4.38	0.00E+00
REACTOME_COLLAGEN_BIOSYNTHESIS_AND_MODIFYING_ENZYMES	4.29	0.00E+00
REACTOME_INTEGRIN_CELL_SURFACE_INTERACTIONS	4.27	0.00E+00
KEGG_LYSOSOME	4.20	0.00E+00
REACTOME_SIGNALING_BY_RECEPTOR_TYROSINE_KINASES	4.16	0.00E+00
REACTOME_DISEASES_OF_GLYCOSYLATION	4.15	0.00E+00
KEGG_CYTOKINE_CYTOKINE_RECEPTOR_INTERACTION	4.13	0.00E+00
REACTOME_ELASTIC_FIBRE_FORMATION	4.00	0.00E+00
REACTOME_INTERLEUKIN_4_AND_INTERLEUKIN_13_SIGNALING	3.95	0.00E+00
REACTOME_METABOLISM_OF_CARBOHYDRATES	3.95	0.00E+00
PID_HIF1_TFPATHWAY	3.95	0.00E+00
KEGG_FOCAL_ADHESION	3.94	0.00E+00
HALLMARK_COAGULATION	3.89	0.00E+00
REACTOME_ASSEMBLY_OF_COLLAGEN_FIBRILS_AND_OTHER_MULTIMERIC_STRUCTURES	3.88	0.00E+00
REACTOME_CHONDROITIN_SULFATE_DERMATAN_SULFATE_METABOLISM	3.85	0.00E+00
REACTOME_NON_INTEGRIN_MEMBRANE_ECM_INTERACTIONS	3.78	0.00E+00
REACTOME_DISEASES_OF_METABOLISM	3.72	0.00E+00
WP_MIRNA_TARGETS_IN_ECM_AND_MEMBRANE_RECEPTORS	3.70	0.00E+00
WP_INFLAMMATORY_RESPONSE_PATHWAY	3.67	0.00E+00

KEGG_MEDICUS_REFERENCE_ITGA_B_FAK_RAC_SIGNALING_PATHWAY	3.64	0.00E+00
REACTOME_NEURONAL_SYSTEM	3.64	0.00E+00
WP_FOCAL_ADHESION_PI3K_AKT_MTOR_SIGNALING_PATHWAY	3.63	0.00E+00
REACTOME_LAMININ_INTERACTIONS	3.63	0.00E+00
REACTOME_NEUTROPHIL_DEGRANULATION	3.61	0.00E+00
PID_INTEGRIN3_PATHWAY	3.59	0.00E+00
REACTOME_MOLECULES_ASSOCIATED_WITH_ELASTIC_FIBRES	3.59	0.00E+00
REACTOME_SIGNALING_BY_GPCR	3.58	0.00E+00
WP_FOCAL_ADHESION	3.57	0.00E+00
HALLMARK_KRAS_SIGNALING_UP	3.56	0.00E+00
PID_UPA_UPAR_PATHWAY	3.55	0.00E+00
REACTOME_PLATELET_ACTIVATION_SIGNALING_AND_AGGREGATION	3.52	0.00E+00
REACTOME_MET_ACTIVATES_PTK2_SIGNALING	3.51	0.00E+00
REACTOME_MET_PROMOTES_CELL_MOTILITY	3.48	0.00E+00
REACTOME_COLLAGEN_DEGRADATION	3.43	0.00E+00
WP_PI3K_AKT_SIGNALING_PATHWAY	3.41	0.00E+00
HALLMARK_GLYCOLYSIS	3.38	0.00E+00
WP_GLYCOSAMINOGLYCAN_SYNTHESIS_IN_FIBROBLASTS	3.37	0.00E+00
REACTOME_SYNDECAN_INTERACTIONS	3.36	0.00E+00
WP_TYPE_I_COLLAGEN_SYNTHESIS_IN_THE_CONTEXT_OF_OSTEOGENESIS_IMPERFECTA	3.36	0.00E+00
KEGG_GLYCOSAMINOGLYCAN_BIOSYNTHESIS_CHONDROITIN_SULFATE	3.36	0.00E+00
REACTOME_BINDING_AND_UPTAKE_OF_LIGANDS_BY_SCAVENGER_RECEPTORS	3.35	0.00E+00
HALLMARK_IL2_STAT5_SIGNALING	3.33	0.00E+00
HALLMARK_APICAL_JUNCTION	3.33	0.00E+00
HALLMARK_P53_PATHWAY	3.30	0.00E+00
REACTOME_SIGNALING_BY_NTRKS	3.30	0.00E+00
WP_INTEGRIN_MEDIATED_CELL_ADHESION	3.30	0.00E+00
HALLMARK_INFLAMMATORY_RESPONSE	3.29	0.00E+00
WP_PLEURAL_MESOTHELIOMA	3.25	0.00E+00
REACTOME_KERATAN_SULFATE_KERATIN_METABOLISM	3.25	0.00E+00
REACTOME_L1CAM_INTERACTIONS	3.20	0.00E+00
WP_REGULATORY_CIRCUITS_OF_THE_STAT3_SIGNALING_PATHWAY	3.20	0.00E+00
REACTOME_RESPONSE_TO_ELEVATED_PLATELET_CYTOSOLIC_CA2	3.19	0.00E+00
KEGG_MEDICUS_REFERENCE_ITGA_B_RHOG_RAC_SIGNALING_PATHWAY	3.19	0.00E+00
HALLMARK_TGF_BETA_SIGNALING	3.17	0.00E+00
REACTOME_SIGNALING_BY_MET	3.12	0.00E+00

KEGG_PATHWAYS_IN_CANCER	3.08	0.00E+00
PID_SYNDECAN_1_PATHWAY	3.06	0.00E+00
HALLMARK_MYOGENESIS	3.05	0.00E+00
WP_BURN_WOUND_HEALING	3.05	0.00E+00
PID_INTEGRIN_CS_PATHWAY	3.04	0.00E+00
KEGG_MEDICUS_REFERENCE_ITGA_B_FAK_CDC42_SIGNALING_PATHWAY	3.02	1.54E-05
PID_VEGF_VEGFR_PATHWAY	3.01	1.52E-05
KEGG_CELL_ADHESION_MOLECULES_CAMS	3.00	1.50E-05
WP_VITAMIN_D_RECEPTOR_PATHWAY	2.99	1.48E-05
REACTOME_O_LINKED_GLYCOSYLATION	2.98	1.46E-05
HALLMARK_IL6_JAK_STAT3_SIGNALING	2.96	1.44E-05
REACTOME_TRANSMISSION_ACROSS_CHEMICAL_SYNAPSES	2.95	4.25E-05
PID_NOTCH_PATHWAY	2.94	4.20E-05
REACTOME_COLLAGEN_CHAIN_TRIMERIZATION	2.94	4.15E-05
WP_PHOTODYNAMIC_THERAPY_INDUCED_HIF_1_SURVIVAL_SIGNALING	2.92	6.85E-05
WP_CALCIUM_REGULATION_IN_CARDIAC_CELLS	2.92	6.77E-05
REACTOME_DISEASES_ASSOCIATED_WITH_GLYCOSAMINOGLYCAN_METABOLISM	2.89	8.03E-05
REACTOME_O_GLYCOSYLATION_OF_TSR_DOMAIN_CONTAINING_PROTEINS	2.89	7.93E-05
REACTOME_DISEASES_ASSOCIATED_WITH_O_GLYCOSYLATION_OF_PROTEINS	2.88	7.84E-05
PID_A6B1_A6B4_INTEGRIN_PATHWAY	2.87	7.75E-05
WP_A_NETWORK_MAP_OF_MACROPHAGE_STIMULATING_PROTEIN_MSP_SIGNALING	2.85	1.15E-04
KEGG_AXON_GUIDANCE	2.84	1.40E-04
REACTOME_NUCLEAR_EVENTS_KINASE_AND_TRANSCRIPTION_FACTOR_ACTIVATION	2.82	1.63E-04
REACTOME_CROSSLINKING_OF_COLLAGEN_FIBRILS	2.82	1.61E-04
REACTOME_HEPARAN_SULFATE_HEPARIN_HS_GAG_METABOLISM	2.82	1.60E-04
HALLMARK_COMPLEMENT	2.82	1.82E-04
REACTOME_CRMPS_IN_SEMA3A_SIGNALING	2.81	2.15E-04
WP_EPITHELIAL_TO_MESENCHYMAL_TRANSITION_IN_COLORECTAL_CANCER	2.80	2.13E-04
KEGG_DILATED_CARDIOMYOPATHY	2.80	2.11E-04
WP_VEGFA_VEGFR2_SIGNALING	2.78	2.32E-04
REACTOME_TRANSPORT_OF_INORGANIC_CATIONS_ANIONS_AND_AMINO_ACIDS_OLIGOPEPTIDES	2.78	2.30E-04
KEGG_MEDICUS_REFERENCE_N_GLYCAN_BIOSYNTHESIS	2.75	3.30E-04
KEGG_HEMATOPOIETIC_CELL_LINEAGE	2.75	3.38E-04

KEGG_MEDICUS_REFERENCE_CHONDROITIN_SULFATE_BIOSYNTHESIS	2.74	3.45E-04
KEGG_MEDICUS_REFERENCE_ITGA_B_RHOGAP_RHOA_SIGNALING_PATHWAY	2.74	3.42E-04
REACTOME_SIGNALLING_TO_ERKS	2.73	3.61E-04
REACTOME_CHONDROITIN_SULFATE_BIOSYNTHESIS	2.73	3.68E-04
KEGG_MEDICUS_REFERENCE_ITGA_B_RHOGEF_RHOA_SIGNALING_PATHWAY	2.72	3.96E-04
REACTOME_DSCAM_INTERACTIONS	2.71	4.45E-04
REACTOME_SIGNALING_BY_VEGF	2.69	5.98E-04
WP_MATRIX_METALLOPROTEINASES	2.69	6.03E-04
PID_AVB3_INTEGRIN_PATHWAY	2.68	6.08E-04
REACTOME_SCAVENGING_BY_CLASS_A_RECEPTORS	2.68	6.23E-04
REACTOME_GPCR_LIGAND_BINDING	2.68	6.17E-04
WP_MIR_509_3P_ALTERATION_OF_YAP1_ECM_AXIS	2.67	6.72E-04
PID_EPHB_FWD_PATHWAY	2.67	6.66E-04
HALLMARK_APOPTOSIS	2.66	6.90E-04
WP_11P11_2_COPY_NUMBER_VARIATION_SYNDROME	2.66	7.23E-04
PID_INTEGRIN4_PATHWAY	2.65	7.66E-04
KEGG_LEUKOCYTE_TRANSENDOTHELIAL_MIGRATION	2.64	7.97E-04
REACTOME_NGF_STIMULATED_TRANSCRIPTION	2.64	8.00E-04
REACTOME_VEGF_LIGAND_RECEPTOR_INTERACTIONS	2.64	8.03E-04
WP_NEUROINFLAMMATION_AND_GLUTAMATERGIC_SIGNALING	2.63	8.15E-04
REACTOME_ACTIVATION_OF_MATRIX_METALLOPROTEINASES	2.62	8.45E-04
WP_CANONICAL_AND_NON_CANONICAL_NOTCH_SIGNALING	2.62	8.74E-04
REACTOME_KERATAN_SULFATE_BIOSYNTHESIS	2.62	8.67E-04
REACTOME_SIGNALLING_TO_RAS	2.62	9.15E-04
HALLMARK_UV_RESPONSE_DN	2.60	1.03E-03
WP_OSTEOBLAST_DIFFERENTIATION_AND_RELATED_DISEASES	2.60	1.08E-03
PID_LYMPH_ANGIOGENESIS_PATHWAY	2.58	1.26E-03
PID_ANGIOPOIETIN_RECEPTOR_PATHWAY	2.58	1.29E-03
WP_EXTRAFOLLICULAR_AND_FOLLICULAR_B_CELL_ACTIVATION_BY_SARS_COV_2	2.57	1.32E-03
PID_EPHA2_FWD_PATHWAY	2.56	1.38E-03
WP_TGF_BETA_RECEPTOR_SIGNALING_IN_SKELETAL_DYSPLASIAS	2.56	1.38E-03
KEGG_MEDICUS_VARIANT_TMPRSS2_ERG_FUSION_TO_TRANSCRIPTIONAL_ACTIVATION	2.56	1.37E-03
REACTOME_PLATELET_AGGREGATION_PLUG_FORMATION	2.55	1.48E-03
WP_ARRHYTHMOGENIC_RIGHT_VENTRICULAR_CARDIOMYOPATHY	2.55	1.48E-03
REACTOME_SLC_MEDIATED_TRANSMEMBRANE_TRANSPORT	2.55	1.49E-03
SA_MMP_CYTOKINE_CONNECTION	2.55	1.51E-03

KEGG_ARRHYTHMOGENIC_RIGHT_VENTRICULAR_CARDIOMYOPATHY_ARVC	2.55	1.54E-03
REACTOME_EPH_EPHRIN_MEDIATED_REPULSION_OF_CELLS	2.55	1.54E-03
BIOCARTA_GHRELIN_PATHWAY	2.54	1.55E-03
REACTOME_A_TETRASACCHARIDE_LINKER_SEQUENCE_IS_REQUIRED_FOR_GAG_SYNTHESIS	2.54	1.63E-03
HALLMARK_ANGIOGENESIS	2.52	1.82E-03
WP_NRP1_TRIGGERED_SIGNALING_PATHWAYS_IN_PANCREATIC_CANCER	2.52	1.86E-03
REACTOME_HS_GAG_DEGRADATION	2.52	1.91E-03
WP_DEGRADATION_PATHWAY_OF_SPHINGOLIPIDS_INCLUDING_DISEASES	2.52	1.89E-03
PID_FAK_PATHWAY	2.52	1.88E-03
WP_GLYCOSAMINOGLYCAN_DEGRADATION	2.51	2.03E-03
KEGG_HYPERTROPHIC_CARDIOMYOPATHY_HCM	2.51	2.14E-03
WP_HIPPO_MERLIN_SIGNALING_DYSREGULATION	2.51	2.13E-03
PID_INTEGRIN5_PATHWAY	2.50	2.19E-03
KEGG_MEDICUS_REFERENCE_RTK_PLCG_ITPR_SIGNALING_PATHWAY	2.50	2.22E-03
PID_NECTIN_PATHWAY	2.49	2.36E-03
REACTOME_RAB_GERANYLGERANYLATION	2.48	2.44E-03
REACTOME_G_ALPHA_Q_SIGNALLING_EVENTS	2.47	2.60E-03
KEGG_JAK_STAT_SIGNALING_PATHWAY	2.47	2.61E-03
REACTOME_NEUROTRANSMITTER_RECEPTORS_AND_POSTSYNAPTIC_SIGNAL_TRANSMISSION	2.46	2.68E-03
WP_PROTEOGLYCAN_BIOSYNTHESIS	2.46	2.69E-03
REACTOME_MATURATION_OF_SARS_COV_1_SPIKE_PROTEIN	2.46	2.69E-03
REACTOME_RUNX1_REGULATES_TRANSCRIPTION_OF_GENES_INVOLVED_IN_DIFFERENTIATION_OF_KERATINOCYTES	2.45	2.97E-03
REACTOME_DEFECTIVE_EXT2_CAUSES_EXOSTOSES_2	2.44	3.02E-03
KEGG_MEDICUS_PATHOGEN_HPV_E6_TO_NOTCH_SIGNALING_PATHWAY_N00380	2.44	3.14E-03
REACTOME_ASPARAGINE_N_LINKED_GLYCOSYLATION	2.44	3.13E-03
REACTOME_GRB2_SOS_PROVIDES_LINKAGE_TO_MAPK_SIGNALING_FOR_INTEGRINS	2.43	3.23E-03
BIOCARTA_MONOCYTE_PATHWAY	2.42	3.42E-03
PID_IL4_2PATHWAY	2.42	3.49E-03
REACTOME_CLASS_B_2_SECRETIN_FAMILY_RECEPTORS	2.41	3.83E-03
REACTOME_ATTACHMENT_AND_ENTRY	2.40	3.98E-03
WP_PRIMARY_FOCAL_SEGMENTAL_GLOMERULOSCLEROSIS_FSGS	2.40	3.98E-03
KEGG_MEDICUS_REFERENCE_ITGA_B_TALIN_VINCULIN_SIGNALING_PATHWAY	2.40	4.09E-03

REACTOME_IMMUNOREGULATORY_INTERACTIONS_BETWEEN_A_LYMPHOID_AND_A_NON_LYMPHOID_CELL	2.40	4.10E-03
REACTOME_LEISHMANIA_INFECTION	2.40	4.14E-03
KEGG_N_GLYCAN_BIOSYNTHESIS	2.39	4.24E-03
WP_NOTCH_SIGNALING_PATHWAY	2.39	4.27E-03
WP_P53_TRANSCRIPTIONAL_GENE_NETWORK	2.39	4.35E-03
KEGG_SMALL_CELL_LUNG_CANCER	2.38	4.43E-03
KEGG_GLYCOSAMINOGLYCAN_DEGRADATION	2.38	4.46E-03
PID_INTEGRIN2_PATHWAY	2.37	4.69E-03
BIOCARTA_NOTCH_PATHWAY	2.37	4.69E-03
WP_IL1_AND_MEGAKARYOCYTES_IN_OBESITY	2.36	4.81E-03
REACTOME_DISSOLUTION_OF_FIBRIN_CLOT	2.36	4.85E-03
BIOCARTA_LYMPHOCYTE_PATHWAY	2.36	4.86E-03
KEGG_GALACTOSE_METABOLISM	2.36	4.94E-03
REACTOME_SIGNALING_BY_NOTCH1	2.34	5.36E-03
REACTOME_INTEGRIN_SIGNALING	2.34	5.56E-03
WP_GLYCOLYSIS_IN_SENESCENCE	2.34	5.54E-03
REACTOME_P130CAS_LINKAGE_TO_MAPK_SIGNALING_FOR_INTEGRINS	2.34	5.66E-03
KEGG_MAPK_SIGNALING_PATHWAY	2.33	5.73E-03
KEGG_GLYCOSAMINOGLYCAN_BIOSYNTHESIS_KERATAN_SULFATE	2.33	5.89E-03
REACTOME_G_ALPHA_12_13_SIGNALLING_EVENTS	2.32	6.25E-03
KEGG_MEDICUS_REFERENCE_IL6_FAMILY_TO_JAK_STAT_SIGNALING_PATHWAY	2.32	6.33E-03
WP_MYOMETRIAL_RELAXATION_AND_CONTRACTION_PATHWAYS	2.32	6.37E-03
WP_SMALL_CELL_LUNG_CANCER	2.31	6.46E-03
WP_HEMATOPOIETIC_STEM_CELL_DIFFERENTIATION	2.31	6.57E-03
REACTOME_POTASSIUM_CHANNELS	2.31	6.57E-03
PID_EPHRINB_REV_PATHWAY	2.31	6.63E-03
BIOCARTA_EICOSANOID_PATHWAY	2.30	6.70E-03
REACTOME_RECYCLING_PATHWAY_OF_L1	2.30	6.94E-03
BIOCARTA_LYM_PATHWAY	2.30	6.97E-03
KEGG_MEDICUS_REFERENCE_CA2_ENTRY_VOLTAGE_GATED_CA2_CHANNEL	2.30	6.96E-03
WP_MARKERS_OF_KIDNEY_CELL_LINEAGE	2.30	6.93E-03
WP_OVERLAP_BETWEEN_SIGNAL_TRANSDUCTION_PATHWAYS_CONTRIBUTING_TO_LMNA_LAMINOPATHIES	2.30	6.94E-03
REACTOME_PRE_NOTCH_EXPRESSION_AND_PROCESSING	2.29	7.08E-03
PID_REELIN_PATHWAY	2.29	7.29E-03
KEGG_MEDICUS_PATHOGEN_HPV_E6_TO_NOTCH_SIGNALING_PATHWAY_N00382	2.28	7.96E-03
REACTOME_SEMAPHORIN_INTERACTIONS	2.27	8.05E-03

REACTOME_RUNX3_REGULATES_P14_ARF	2.27	8.13E-03
WP_NCRNAS_INVOLVED_IN_STAT3_SIGNALING_IN_HEPATOCELLULAR_CARCINOMA	2.26	8.59E-03
REACTOME_LGI_ADAM_INTERACTIONS	2.26	8.60E-03
PID_FRA_PATHWAY	2.26	8.62E-03
REACTOME_CALNEXIN_CALRETICULIN_CYCLE	2.26	8.59E-03
WP_FRAGILE_X_SYNDROME	2.26	8.65E-03
REACTOME_SIGNALING_BY_NOTCH2	2.25	9.44E-03
KEGG_MEDICUS_VARIANT_MUTATION_CAUSED_ABERRANT_ABETA_TO_ANTEROGRADE_AXONAL_TRANSPORT	2.24	9.85E-03
REACTOME_DISEASES_ASSOCIATED_WITH_GLYCOSYLATION_PRECURSOR_BIOSYNTHESIS	2.23	1.02E-02
WP_MAPK_SIGNALING_PATHWAY	2.23	1.03E-02
REACTOME_DAP12_INTERACTIONS	2.23	1.03E-02
KEGG_BASAL_CELL_CARCINOMA	2.23	1.04E-02
REACTOME_ACTIVATED_NOTCH1_TRANSMITS_SIGNAL_TO_THE_NUCLEUS	2.23	1.04E-02
WP_N_GLYCAN_BIOSYNTHESIS	2.22	1.06E-02
REACTOME_SIGNALING_BY_INTERLEUKINS	2.22	1.06E-02
REACTOME_MATURATION_OF_SARS_COV_2_SPIKE_PROTEIN	2.22	1.06E-02
KEGG_MEDICUS_REFERENCE_IGG_FCGR_RAC_SIGNALING_PATHWAY	2.22	1.11E-02
BIOCARTA_BARRESTIN_PATHWAY	2.21	1.13E-02
KEGG_MEDICUS_REFERENCE_WNT_SIGNALING_MODULATION_SOST_LRP4	2.21	1.13E-02
REACTOME_EPHRIN_SIGNALING	2.21	1.13E-02
BIOCARTA_RECK_PATHWAY	2.21	1.15E-02
REACTOME_SPHINGOLIPID_METABOLISM	2.21	1.14E-02
PID_ENDOTHELIN_PATHWAY	2.21	1.16E-02
REACTOME_ANCHORING_FIBRIL_FORMATION	2.20	1.19E-02
REACTOME_HS_GAG_BIOSYNTHESIS	2.20	1.20E-02
WP_ENDOCHONDRAL_OSSIFICATION	2.20	1.20E-02
REACTOME_TRANSPORT_OF_SMALL_MOLECULES	2.20	1.21E-02
WP_METABOLIC_PATHWAYS_OF_FIBROBLASTS	2.20	1.21E-02
REACTOME_GLYCOSPHINGOLIPID_METABOLISM	2.20	1.23E-02
REACTOME_INTERLEUKIN_27_SIGNALING	2.19	1.26E-02
WP_SPHINGOLIPID_METABOLISM_INTEGRATED_PATHWAY	2.19	1.28E-02
WP_HAIR_FOLLICLE_DEVELOPMENT_CYTODIFFERENTIATION_PART_3_OF_3	2.18	1.30E-02
WP_HEPATITIS_C_AND_HEPATOCELLULAR_CARCINOMA	2.18	1.31E-02
REACTOME_HYALURONAN_UPTAKE_AND_DEGRADATION	2.18	1.32E-02
KEGG_LEISHMANIA_INFECTION	2.18	1.33E-02

KEGG_MEDICUS_REFERENCE_GF_RTK_RAS_ERK_SIGNALING_PATHWAY	2.18	1.33E-02
REACTOME_IRE1ALPHA_ACTIVATES_CHAPERONES	2.18	1.35E-02
REACTOME_LATE_SARS_COV_2_INFECTION_EVENTS	2.18	1.35E-02
WP_CKAP4_SIGNALING_PATHWAY_MAP	2.18	1.35E-02
REACTOME_SIGNALING_BY_TGFB_FAMILY_MEMBERS	2.17	1.36E-02
PID_PI3K_PLC_TRK_PATHWAY	2.17	1.39E-02
PID_INTEGRIN_A9B1_PATHWAY	2.17	1.39E-02
REACTOME_REGULATION_OF_CYTOSKELETAL_REMODELING_AND_CELL_SPREADING_BY_IPP_COMPLEX_COMPONENTS	2.17	1.40E-02
PID_PDGFRA_PATHWAY	2.16	1.42E-02
REACTOME_OTHER_SEMAPHORIN_INTERACTIONS	2.16	1.42E-02
WP_PROSTAGLANDIN_AND_LEUKOTRIENE_METABOLISM_IN_SENESCENCE_WP5321	2.16	1.43E-02
REACTOME_PHOSPHATE_BOND_HYDROLYSIS_BY_NTPDASE_PROTEINS	2.16	1.46E-02
WP_COMPLEMENT_AND_COAGULATION_CASCADES	2.16	1.47E-02
REACTOME_OTHER_INTERLEUKIN_SIGNALING	2.16	1.46E-02
WP_NCRNAS_INVOLVED_IN_WNT_SIGNALING_IN_HEPATOCELLULAR_CARCINOMA	2.15	1.47E-02
WP_ESTROGEN_RECEPTOR_PATHWAY	2.15	1.48E-02
WP_INSULIN_SIGNALING	2.15	1.51E-02
WP_ENDOCHONDRAL_OSSIFICATION_WITH_SKELETAL_DYSPLASIAS	2.15	1.53E-02
REACTOME_INTERLEUKIN_6_FAMILY_SIGNALING	2.15	1.53E-02
WP_GLUCOSE_METABOLISM_IN_TRIPLE_NEGATIVE_BREAST_CANCER_CELLS	2.15	1.55E-02
REACTOME_INTERACTION_BETWEEN_L1_AND_ANKYRINS	2.14	1.56E-02
REACTOME_ION_CHANNEL_TRANSPORT	2.14	1.59E-02
WP_PROLACTIN_SIGNALING_PATHWAY	2.14	1.59E-02
REACTOME_NCAM1_INTERACTIONS	2.14	1.60E-02
REACTOME_SIGNALING_BY_NOTCH1_T_7_9_NOTCH1_M1580_K2555_TRANSLOCATION_MUTANT	2.14	1.60E-02
KEGG_MEDICUS_REFERENCE_REGULATION_OF_FIBRINOLYTIC_SYSTEM_C1INH	2.14	1.60E-02
REACTOME_LYSOSPHINGOLIPID_AND_LPA_RECEPTORS	2.13	1.62E-02
REACTOME_SARS_COV_1_TARGETS_HOST_INTRACELLULAR_SIGNALLING_AND_REGULATORY_PATHWAYS	2.13	1.63E-02
KEGG_MEDICUS_REFERENCE_GLYCOLYSIS	2.13	1.63E-02
PID_P53_DOWNSTREAM_PATHWAY	2.13	1.66E-02
HALLMARK_HEDGEHOG_SIGNALING	2.12	1.73E-02
BIOCARTA_HDAC_PATHWAY	2.12	1.72E-02
WP_PROSTAGLANDIN_SYNTHESIS_AND_REGULATION	2.12	1.73E-02

REACTOME_EPH_EPHRIN_SIGNALING	2.12	1.72E-02
KEGG_MEDICUS_REFERENCE_REGULATION_OF_FIBRINOLYTIC_SYSTEM_PAI	2.12	1.72E-02
PID_AMB2_NEUTROPHILS_PATHWAY	2.11	1.76E-02
KEGG_NATURAL_KILLER_CELL_MEDIATED_CYTOTOXICITY	2.11	1.80E-02
KEGG_MEDICUS_PATHOGEN_HCMV_GH_TO_ITGA_B_RHOA_SIGNALING_PATHWAY	2.11	1.81E-02
WP_AMPLIFICATION_AND_EXPANSION_OF_ONCOGENIC_PATHWAYS_AS_METASTATIC_TRAITS	2.11	1.82E-02
WP_CHEMOKINE_SIGNALING_PATHWAY	2.11	1.84E-02
KEGG_MEDICUS_REFERENCE_NOTCH_PROTEOLYTIC_ACTIVATION	2.10	1.90E-02
BIOCARTA_PLATELETAPP_PATHWAY	2.10	1.89E-02
KEGG_GLYCOSPHINGOLIPID_BIOSYNTHESIS_GANGLIO_SERIES	2.10	1.89E-02
REACTOME_AMINO_ACID_TRANSPORT_ACROSS_THE_PLASMA_MEMBRANE	2.10	1.89E-02
WP_COPPER_HOMEOSTASIS	2.10	1.89E-02
REACTOME_KERATAN_SULFATE_DEGRADATION	2.10	1.89E-02
WP_SMALL_LIGAND_GPCRS	2.10	1.89E-02
REACTOME_INTERLEUKIN_15_SIGNALING	2.10	1.88E-02
WP_GANGLIO_SERIES_SPHINGOLIPID_METABOLISM	2.10	1.89E-02
WP_GPCRS_CLASS_A_RHODOPSIN_LIKE	2.10	1.88E-02
KEGG_MEDICUS_REFERENCE_GF_RTK_RAS_PI3K_SIGNALING_PATHWAY	2.09	1.91E-02
PID_AVB3_OPN_PATHWAY	2.09	1.92E-02
REACTOME_ARMS_MEDIATED_ACTIVATION	2.09	1.95E-02
REACTOME_POTENTIAL_THERAPEUTICS_FOR_SARS	2.09	1.95E-02
WP_PHOTODYNAMIC_THERAPY_INDUCED_NF_KB_SURVIVAL_SIGNALING	2.09	1.95E-02
REACTOME_RHOA_GTPASE_CYCLE	2.08	2.02E-02
WP_NOTCH_SIGNALING	2.08	2.04E-02
WP_EMBRYONIC_STEM_CELL_PLURIPOTENCY_PATHWAYS	2.08	2.08E-02
REACTOME_N_GLYCAN_ANTENNAE_ELONGATION_IN_THE_MEDIAL_TRANS_GOLGI	2.07	2.12E-02
BIOCARTA_PS1_PATHWAY	2.07	2.13E-02
REACTOME_DEFECTIVE_LFNG_CAUSES_SCDO3	2.07	2.16E-02
PID_HIF2PATHWAY	2.07	2.17E-02
PID_WNT_SIGNALING_PATHWAY	2.07	2.16E-02
REACTOME_PROTEIN_PROTEIN_INTERACTIONS_AT_SYNAPSES	2.07	2.18E-02
PID_ALK1_PATHWAY	2.06	2.18E-02
KEGG_MEDICUS_VARIANT_NOTCH_OVEREXPRESSION_TO_NOTCH_SIGNALING_PATHWAY	2.06	2.18E-02
REACTOME_INDUCTION_OF_CELL_CELL_FUSION	2.06	2.23E-02
PID_CXCR4_PATHWAY	2.06	2.23E-02

WP_WNT_SIGNALING	2.06	2.23E-02
WP_GLYCOLYSIS_AND_GLUCONEOGENESIS	2.06	2.23E-02
PID_ER_NONGENOMIC_PATHWAY	2.06	2.23E-02
WP_TH17_CELL_DIFFERENTIATION_PATHWAY	2.06	2.23E-02
REACTOME_SEMA3A_PAK_DEPENDENT_AXON_REPULSION	2.05	2.29E-02
KEGG_INTESTINAL_IMMUNE_NETWORK_FOR_IGA_PRODUCTION	2.05	2.28E-02
REACTOME_TNFS_BIND_THEIR_PHYSIOLOGICAL_RECEPTORS	2.05	2.30E-02
WP_ONCOSTATIN_M_SIGNALING_PATHWAY	2.05	2.31E-02
REACTOME_CELL_CELL_COMMUNICATION	2.05	2.33E-02
KEGG_NOTCH_SIGNALING_PATHWAY	2.05	2.37E-02
KEGG_MEDICUS_PATHOGEN_HPV_E6_TO_NOTCH_SIGNALING_PATHWAY_N00381	2.04	2.37E-02
WP_PARKINSON_39_S_DISEASE_PATHWAY	2.04	2.41E-02
REACTOME_CELL_DEATH_SIGNALLING_VIA_NRAGE_NRIF_AND_NADE	2.04	2.44E-02
WP_COMPLEMENT_SYSTEM_IN_NEURONAL_DEVELOPMENT_AND_PLASTICITY	2.04	2.44E-02
REACTOME_N_GLYCAN_ANTENNAE_ELONGATION	2.04	2.46E-02
HALLMARK_WNT_BETA_CATENIN_SIGNALING	2.03	2.52E-02
REACTOME_TGF_BETA_RECEPTOR_SIGNALING_ACTIVATES_SMADS	2.03	2.52E-02
REACTOME_G_ALPHA_I_SIGNALLING_EVENTS	2.03	2.52E-02
PID_IL6_7_PATHWAY	2.03	2.51E-02
REACTOME_RESPONSE_TO_METAL_IONS	2.03	2.54E-02
REACTOME_METALLOTHIONEINS_BIND_METALS	2.03	2.54E-02
KEGG_COMPLEMENT_AND_COAGULATION_CASCADES	2.03	2.54E-02
WP_COMPLEMENT_SYSTEM	2.03	2.57E-02
HALLMARK_UV_RESPONSE_UP	2.03	2.57E-02
KEGG_MEDICUS_REFERENCE_CARDIAC_TYPE_VGCC_RYR_SIGNALING	2.02	2.65E-02
WP_AEROBIC_GLYCOLYSIS	2.02	2.64E-02
REACTOME_CA2_ACTIVATED_K_CHANNELS	2.02	2.71E-02
WP_NEOVASCULARISATION_PROCESSES	2.01	2.75E-02
WP_NUCLEAR_RECEPTORS_META_PATHWAY	2.01	2.74E-02
KEGG_MEDICUS_REFERENCE_SKELETAL_TYPE_VGCC_RYR_SIGNALING	2.01	2.79E-02
WP_PLURIPOTENT_STEM_CELL_DIFFERENTIATION_PATHWAY	2.01	2.83E-02
REACTOME_ABERRANT_REGULATION_OF_MITOTIC_G1_S_TRANSITION_IN_CANCER_DUE_TO_RB1_DEFECTS	2.01	2.85E-02
HALLMARK_ESTROGEN_RESPONSE_LATE	2.00	2.87E-02
KEGG_MEDICUS_REFERENCE_KERATAN_SULFATE_DEGRADATION	2.00	2.89E-02
WP_TGF_BETA_RECEPTOR_SIGNALING	2.00	2.92E-02
REACTOME_SIGNALING_BY_BMP	2.00	2.92E-02

WP_EBOLA_VIRUS_INFECTION_IN_HOST	2.00	2.95E-02
REACTOME_NCAM_SIGNALING_FOR_NEURITE_OUT_GROWTH	2.00	2.97E-02
WP_PHOTODYNAMIC_THERAPY_INDUCED_AP_1_SURVIVAL_SIGNALING	2.00	2.96E-02
PID_SYNDECAN_4_PATHWAY	1.99	3.01E-02
REACTOME_PLASMA_LIPOPROTEIN_REMODELING	1.99	3.02E-02
REACTOME_SIGNALING_BY_NOTCH1_PEST_DOMAIN_MUTANTS_IN_CANCER	1.99	3.02E-02
REACTOME_NRAGE_SIGNALS_DEATH_THROUGH_JNK	1.99	3.03E-02
REACTOME_TRANSLATION_OF_SARS_COV_1_STRUCTURAL_PROTEINS	1.99	3.07E-02
REACTOME_PRE_NOTCH_PROCESSING_IN_GOLGI	1.99	3.11E-02
WP_GENES_ASSOCIATED_WITH_THE_DEVELOPMENT_OF_RHEUMATOID_ARTHRITIS	1.98	3.15E-02
REACTOME_GPER1_SIGNALING	1.98	3.19E-02
WP_IL_24_SIGNALING_PATHWAY	1.98	3.18E-02
REACTOME_CARDIAC_CONDUCTION	1.98	3.23E-02
KEGG_MEDICUS_REFERENCE_WNT5A_ROR_SIGNALING_PATHWAY	1.97	3.25E-02
PID_GLYPICAN_1PATHWAY	1.97	3.26E-02
REACTOME_SYNTHESIS_OF_SUBSTRATES_IN_N_GLYCAN_BIOSYTHESIS	1.97	3.32E-02
PID_PTP1B_PATHWAY	1.97	3.33E-02
REACTOME_ANTI_INFLAMMATORY_RESPONSE_FAVOURING_LEISHMANIA_PARASITE_INFECTION	1.97	3.34E-02
REACTOME_SIGNALING_BY_PDGF	1.97	3.34E-02
KEGG_GLYCOSPHINGOLIPID_BIOSYNTHESIS_GLOBO_SERIES	1.97	3.38E-02
REACTOME_SENSORY_PERCEPTION	1.96	3.38E-02
REACTOME_CHL1_INTERACTIONS	1.96	3.40E-02
WP_CANONICAL_AND_NON_CANONICAL_TGF_B_SIGNALING	1.96	3.43E-02
WP_PROSTAGLANDIN_AND_LEUKOTRIENE_METABOLISM_IN_SENESCENCE_WP5122	1.96	3.54E-02
REACTOME_MUCOPOLYSACCHARIDOSES	1.96	3.53E-02
REACTOME_SIGNALING_BY_PDGFRA_TRANSMEMBRANE_JUXTAMEMBRANE_AND_KINASE_DOMAIN_MUTANTS	1.95	3.56E-02
REACTOME_CLASS_A_1_RHODOPSIN_LIKE_RECEPTORS	1.95	3.57E-02
KEGG_GNRH_SIGNALING_PATHWAY	1.95	3.57E-02
REACTOME_ION_HOMEOSTASIS	1.95	3.61E-02
REACTOME_WNT_LIGAND_BIOGENESIS_AND_TRAFFICKING	1.95	3.64E-02
REACTOME_SIGNALING_BY_NOTCH3	1.94	3.76E-02
KEGG_MEDICUS_VARIANT_LRP6_OVEREXPRESSION_TO_WNT_SIGNALING_PATHWAY	1.94	3.80E-02
REACTOME_O_LINKED_GLYCOSYLATION_OF_MUCINS	1.94	3.80E-02
REACTOME_SIGNALING_BY_ERBB4	1.94	3.79E-02

KEGG_REGULATION_OF_ACTIN_CYTOSKELETON	1.94	3.80E-02
REACTOME_FGFRL1_MODULATION_OF_FGFR1_SIGNALING	1.94	3.80E-02
REACTOME_NUCLEAR_SIGNALING_BY_ERBB4	1.94	3.82E-02
REACTOME_GLYCOSPHINGOLIPID_BIOSYNTHESIS	1.94	3.81E-02
REACTOME_IL_6_TYPE_CYTOKINE_RECEPTOR_LIGAND_INTERACTIONS	1.94	3.84E-02
WP_ROBO4_AND_VEGF_SIGNALING_PATHWAYS_CROSSTALK	1.93	3.88E-02
WP_PURINERGIC_SIGNALING	1.93	3.93E-02
PID_TRKR_PATHWAY	1.93	3.95E-02
WP_TYROBP_CAUSAL_NETWORK_IN_MICROGLIA	1.93	3.95E-02
BIOCARTA_P53_PATHWAY	1.93	3.98E-02
WP_PHYSIOLOGICAL_AND_PATHOLOGICAL_HYPERTROPHY_OF_THE_HEART	1.93	3.98E-02
KEGG_MEDICUS_VARIANT_EWSR1_WT1_FUSION_TO_TRANSCRIPTIONAL_ACTIVATION	1.93	3.98E-02
WP_RAC1_PAK1_P38_MMP2_PATHWAY	1.93	4.01E-02
WP_BREAST_CANCER_PATHWAY	1.92	4.04E-02
WP_1P36_COPY_NUMBER_VARIATION_SYNDROME	1.92	4.06E-02
WP_16P11_2_DISTAL_DELETION_SYNDROME	1.92	4.17E-02
WP_SYNTHESIS_OF_CERAMIDES_AND_1_DEOXYCERAMIDES	1.92	4.19E-02
REACTOME_PLATELET_HOMEOSTASIS	1.91	4.33E-02
REACTOME_MUSCLE_CONTRACTION	1.91	4.33E-02
REACTOME_SHC_RELATED_EVENTS_TRIGGERED_BY_IGF1R	1.91	4.35E-02
WP_LNCRNA_IN_CANONICAL_WNT_SIGNALING_AND_COLORECTAL_CANCER	1.91	4.35E-02
WP_SELENIUM_MICRONUTRIENT_NETWORK	1.91	4.37E-02
REACTOME_NETRIN_1_SIGNALING	1.91	4.38E-02
HALLMARK_ESTROGEN_RESPONSE_EARLY	1.91	4.38E-02
KEGG_CHEMOKINE_SIGNALING_PATHWAY	1.90	4.39E-02
REACTOME_AMYLOID_FIBER_FORMATION	1.90	4.45E-02
REACTOME_SIGNALING_BY_NOTCH	1.90	4.45E-02
WP_NUCLEOTIDE_GPCRS	1.90	4.44E-02
KEGG_MEDICUS_REFERENCE_CYTOKINE_JAK_STAT_SIGNALING_PATHWAY	1.90	4.45E-02
WP_INFLUENCE_OF_LAMINOPATHIES_ON_WNT_SIGNALING	1.89	4.62E-02
PID_AP1_PATHWAY	1.89	4.62E-02
WP_GPCRS_OTHER	1.89	4.60E-02
REACTOME_ACTIVATED_NTRK3_SIGNALS_THROUGH_RAS	1.89	4.65E-02
BIOCARTA_MTA3_PATHWAY	1.89	4.64E-02
REACTOME_MAPK_FAMILY_SIGNALING_CASCADES	1.89	4.65E-02
WP_NANOPARTICLE_MEDIATED_ACTIVATION_OF_RECEPTOR_SIGNALING	1.89	4.66E-02

REACTOME_SIGNALING_BY_NTRK2_TRKB	1.89	4.66E-02
REACTOME_N_GLYCAN_TRIMMING_IN_THE_ER_AND_CALNEXIN_CALRETICULIN_CYCLE	1.89	4.72E-02
WP_SPINAL_CORD_INJURY	1.88	4.74E-02
REACTOME_N_GLYCAN_TRIMMING_AND_ELONGATION_IN_THE_CIS_GOLGI	1.88	4.87E-02
WP_ZINC_HOMEOSTASIS	1.88	4.89E-02
BIOCARTA_GRANULOCYTES_PATHWAY	1.88	4.90E-02
KEGG_MEDICUS_REFERENCE_NOTCH_SIGNALING_PATHWAY	1.88	4.89E-02
KEGG_MEDICUS_REFERENCE_TRANSLATION_INITIATION	-7.73	0.00E+00
REACTOME_EUKARYOTIC_TRANSLATION_ELONGATION	-7.58	0.00E+00
REACTOME_RESPONSE_OF_EIF2AK4_GCN2_TO_AMINO_ACID_DEFICIENCY	-7.55	0.00E+00
REACTOME_EUKARYOTIC_TRANSLATION_INITIATION	-7.39	0.00E+00
WP_CYTOPLASMIC_RIBOSOMAL_PROTEINS	-7.33	0.00E+00
KEGG_RIBOSOME	-7.32	0.00E+00
REACTOME_RRNA_PROCESSING	-7.20	0.00E+00
REACTOME_SELENOAMINO_ACID_METABOLISM	-7.00	0.00E+00
REACTOME_NONSENSE_MEDIATED_DECAY_NMD	-6.81	0.00E+00
REACTOME_TRANSLATION	-6.74	0.00E+00
REACTOME_SRP_DEPENDENT_COTRANSLATIONAL_PROTEIN_TARGETING_TO_MEMBRANE	-6.60	0.00E+00
REACTOME_INFLUENZA_INFECTION	-6.54	0.00E+00
REACTOME_REGULATION_OF_EXPRESSION_OF_SLITS_AND_ROBOS	-6.00	0.00E+00
REACTOME_CELLULAR_RESPONSE_TO_STARVATION	-5.89	0.00E+00
REACTOME_METABOLISM_OF_AMINO_ACIDS_AND_DERIVATIVES	-5.78	0.00E+00
HALLMARK_MYC_TARGETS_V1	-5.63	0.00E+00
REACTOME_SIGNALING_BY_ROBO_RECEPTORS	-5.11	0.00E+00
REACTOME_SARS_COV_1_MODULATES_HOST_TRANSLATION_MACHINERY	-4.85	0.00E+00
KEGG_MEDICUS_PATHOGEN_SARS_COV_2_NSP1_TO_TRANSLATION_INITIATION	-4.80	0.00E+00
REACTOME_ACTIVATION_OF_THE_MRNA_UPON_BINDING_OF_THE_CAP_BINDING_COMPLEX_AND_EIFS_AND_SUBSEQUENT_BINDING_TO_43S	-4.73	0.00E+00
REACTOME_CELL_CYCLE_MITOTIC	-4.68	0.00E+00
REACTOME_SARS_COV_2_HOST_INTERACTIONS	-4.66	0.00E+00
REACTOME_M_PHASE	-4.62	0.00E+00
HALLMARK_E2F_TARGETS	-4.61	0.00E+00
REACTOME_SARS_COV_2_MODULATES_HOST_TRANSLATION_MACHINERY	-4.54	0.00E+00
REACTOME_CELL_CYCLE_CHECKPOINTS	-4.03	0.00E+00
REACTOME_HIV_INFECTION	-3.97	0.00E+00

REACTOME_PROCESSING_OF_CAPPED_INTRON_CONTAINING_PRE_MRNA	-3.96	0.00E+00
REACTOME_NERVOUS_SYSTEM_DEVELOPMENT	-3.90	0.00E+00
HALLMARK_MYC_TARGETS_V2	-3.88	0.00E+00
WP_CHOLESTEROL_METABOLISM_WITH_BLOCH_AND_KANDUTSCH_RUSSELL_PATHWAYS	-3.87	0.00E+00
REACTOME_DNA_REPAIR	-3.86	0.00E+00
HALLMARK_OXIDATIVE_PHOSPHORYLATION	-3.82	0.00E+00
REACTOME_MITOTIC_PROMETAPHASE	-3.80	0.00E+00
REACTOME_MITOTIC_METAPHASE_AND_ANAPHASE	-3.76	0.00E+00
REACTOME_SARS_COV_1_HOST_INTERACTIONS	-3.74	0.00E+00
HALLMARK_MTORC1_SIGNALING	-3.69	0.00E+00
REACTOME_RRNA_MODIFICATION_IN_THE_NUCLEUS_AND_CYTOSOL	-3.56	0.00E+00
REACTOME_SEPARATION_OF_SISTER_CHROMATIDS	-3.56	0.00E+00
REACTOME_HIV_LIFE_CYCLE	-3.43	0.00E+00
REACTOME_NUCLEAR_EVENTS_MEDIATED_BY_NFE2L2	-3.43	0.00E+00
REACTOME_UB_SPECIFIC_PROCESSING_PROTEASES	-3.41	0.00E+00
WP_DNA_IR_DAMAGE_AND_CELLULAR_RESPONSE_VIA_ATR	-3.39	0.00E+00
REACTOME_CHOLESTEROL_BIOSYNTHESIS	-3.38	0.00E+00
REACTOME_SARS_COV_INFECTIONS	-3.37	0.00E+00
WP_CHOLESTEROL_BIOSYNTHESIS_PATHWAY_IN_HEPATOCYTES	-3.30	0.00E+00
REACTOME_DEUBIQUITINATION	-3.30	0.00E+00
REACTOME_MITOCHONDRIAL_TRANSLATION	-3.27	0.00E+00
PID_MYC_ACTIV_PATHWAY	-3.25	0.00E+00
WP_AMINO_ACID_METABOLISM	-3.25	0.00E+00
WP_CHOLESTEROL_BIOSYNTHESIS_PATHWAY	-3.24	0.00E+00
HALLMARK_MITOTIC_SPINDLE	-3.24	0.00E+00
REACTOME_MRNA_SPLICING	-3.24	0.00E+00
WP_ELECTRON_TRANSPORT_CHAIN_OXPHOS_SYSTEM_IN_MITOCHONDRIA	-3.22	0.00E+00
KEGG_HUNTINGTONS_DISEASE	-3.20	0.00E+00
REACTOME_REGULATION_OF_MRNA_STABILITY_BY_PROTEINS_THAT_BIND_AU_RICH_ELEMENTS	-3.20	0.00E+00
REACTOME_ORGANELLE_BIOGENESIS_AND_MAINTENANCE	-3.19	0.00E+00
REACTOME_SARS_COV_2_INFECTION	-3.17	0.00E+00
KEGG_OXIDATIVE_PHOSPHORYLATION	-3.17	0.00E+00
REACTOME_RESPIRATORY_ELECTRON_TRANSPORT_ATP_SYNTHESIS_BY_CHEMIOSMOTIC_COUPLING_AND_HEAT_PRODUCTION_BY_UNCOUPLING_PROTEINS	-3.16	0.00E+00
WP_ENTEROCYTE_CHOLESTEROL_METABOLISM	-3.16	0.00E+00
PID_AURORA_B_PATHWAY	-3.15	0.00E+00

WP_STEROL_REGULATORY_ELEMENT_BINDING_PROTEINS_SREBP_SIGNALING	-3.13	1.42E-05
REACTOME_MITOTIC_G2_G2_M_PHASES	-3.12	2.75E-05
REACTOME_METABOLISM_OF_POLYAMINES	-3.12	2.71E-05
KEGG_MEDICUS_REFERENCE_ASSEMBLY_AND_TRAFFICKING_OF_TELOMERASE	-3.11	3.97E-05
REACTOME_THE_CITRIC_ACID_TCA_CYCLE_AND_RESPIRATORY_ELECTRON_TRANSPORT	-3.08	3.91E-05
REACTOME_DNA_DOUBLE_STRAND_BREAK_REPAIR	-3.07	3.86E-05
REACTOME_KEAP1_NFE2L2_PATHWAY	-3.06	3.80E-05
REACTOME_ACTIVATION_OF_GENE_EXPRESSION_BY_SREBF_SREBP	-3.05	4.98E-05
REACTOME_TRNA_AMINOACYLATION	-3.04	4.91E-05
REACTOME_STABILIZATION_OF_P53	-3.04	4.84E-05
HALLMARK_G2M_CHECKPOINT	-3.03	5.96E-05
REACTOME_UCH_PROTEINASES	-3.00	5.88E-05
KEGG_PARKINSONS_DISEASE	-2.98	5.80E-05
REACTOME_REGULATION_OF_CHOLESTEROL_BIOSYNTHESIS_BY_SREBP_SREBF	-2.97	5.73E-05
HALLMARK_CHOLESTEROL_HOMEOSTASIS	-2.95	9.03E-05
REACTOME_APC_C_MEDIATED_DEGRADATION_OF_CELL_CYCLE_PROTEINS	-2.92	1.00E-04
REACTOME_FCERI_MEDIATED_NF_KB_ACTIVATION	-2.92	9.91E-05
WP_DNA_REPAIR_PATHWAYS_FULL_NETWORK	-2.91	1.09E-04
KEGG_MEDICUS_VARIANT_SCRAPIE_CONFORMATION_PRPSC_TO_26S_PROTEASOME_MEDIATED_PROTEIN_DEGRADATION	-2.91	1.07E-04
WP_CHOLESTEROL_SYNTHESIS_DISORDERS	-2.90	1.17E-04
REACTOME_NEGATIVE_REGULATION_OF_NOTCH4_SIGNALING	-2.90	1.16E-04
REACTOME_TRNA_PROCESSING	-2.90	1.14E-04
REACTOME_CELLULAR_RESPONSE_TO_CHEMICAL_STRESS	-2.89	1.23E-04
REACTOME_TRANSCRIPTIONAL_REGULATION_BY_TP53	-2.88	1.22E-04
REACTOME_S_PHASE	-2.88	1.20E-04
REACTOME_REGULATION_OF_TP53_ACTIVITY	-2.88	1.19E-04
KEGG_SPLICEOSOME	-2.87	1.18E-04
REACTOME_HOST_INTERACTIONS_OF_HIV_FACTORS	-2.86	1.26E-04
REACTOME_TRANSCRIPTION_COUPLED_NUCLEOTIDE_EXCISION_REPAIR_TC_NER	-2.86	1.34E-04
REACTOME_HOMOLOGY_DIRECTED_REPAIR	-2.85	1.42E-04
KEGG_PROTEASOME	-2.84	1.50E-04
KEGG_MEDICUS_VARIANT_MUTATION_CAUSED_ABERRANT_SNCA_TO_26S_PROTEASOME_MEDIATED_PROTEIN_DEGRADATION	-2.82	1.86E-04
REACTOME_RESPIRATORY_ELECTRON_TRANSPORT	-2.82	1.84E-04
REACTOME_CHROMOSOME_MAINTENANCE	-2.82	1.82E-04
REACTOME_AUF1_HNRNP_D0_BINDS_AND_DESTABILIZES_MRNA	-2.82	1.80E-04

REACTOME_DEGRADATION_OF_AXIN	-2.82	1.87E-04
REACTOME_NUCLEOTIDE_EXCISION_REPAIR	-2.80	2.21E-04
REACTOME_DECTIN_1_MEDIATED_NONCANONICAL_NF_KB_SIGNALING	-2.79	2.36E-04
REACTOME_G2_M_CHECKPOINTS	-2.79	2.51E-04
REACTOME_FORMATION_OF_TC_NER_PRE_INCISION_COMPLEX	-2.77	2.74E-04
REACTOME_REGULATION_OF_PTEN_STABILITY_AND_ACTIVITY	-2.76	2.80E-04
REACTOME_FOLDING_OF_ACTIN_BY_CCT_TRIC	-2.76	2.78E-04
REACTOME_DEGRADATION_OF_DVL	-2.75	2.75E-04
REACTOME_GLOBAL_GENOME_NUCLEOTIDE_EXCISION_REPAIR_GG_NER	-2.75	2.81E-04
REACTOME_CROSS_PRESENTATION_OF_SOLUBLE_EXOGENOUS_ANTIGENS_ENDOSOMES	-2.75	2.78E-04
REACTOME_SCF_BETA_TRCP_MEDIATED_DEGRADATION_OF_EMI1	-2.75	2.75E-04
REACTOME_MITOTIC_SPINDLE_CHECKPOINT	-2.73	3.29E-04
REACTOME_RNA_POLYMERASE_I_TRANSCRIPTION_INITIATION	-2.73	3.42E-04
REACTOME_RHOBTB_GTPASE_CYCLE	-2.72	3.55E-04
WP_NONALCOHOLIC_FATTY_LIVER_DISEASE	-2.70	4.22E-04
REACTOME_RESOLUTION_OF_SISTER_CHROMATID_COHESION	-2.70	4.49E-04
WP_TRANSLATION_FACTORS	-2.69	4.60E-04
REACTOME_MITOCHONDRIAL_TRNA_AMINOACYLATION	-2.69	4.56E-04
REACTOME_SUMOYLATION	-2.68	4.83E-04
WP_MRNA_PROCESSING	-2.68	5.01E-04
KEGG_MEDICUS_VARIANT_MUTATION_CAUSED_ABERRANT_ABETA_TO_26S_PROTEASOME_MEDIATED_PROTEIN_DEGRADATION	-2.68	5.11E-04
KEGG_MEDICUS_REFERENCE_26S_PROTEASOME_MEDIATED_PROTEIN_DEGRADATION	-2.67	5.51E-04
KEGG_MEDICUS_VARIANT_MUTATION_CAUSED_ABERRANT_HTT_TO_26S_PROTEASOME_MEDIATED_PROTEIN_DEGRADATION	-2.67	5.46E-04
REACTOME_RNA_POLYMERASE_I_TRANSCRIPTION	-2.67	5.42E-04
KEGG_MEDICUS_VARIANT_MUTATION_CAUSED_ABERRANT_SOD1_TO_26S_PROTEASOME_MEDIATED_PROTEIN_DEGRADATION	-2.65	5.95E-04
REACTOME_MITOTIC_PROPHASE	-2.65	5.91E-04
WP_GENES_RELATED_TO_PRIMARY_CILIUM_DEVELOPMENT_BASED_ON_CRISPR	-2.65	5.93E-04
KEGG_GLYOXYLATE_AND_DICARBOXYLATE_METABOLISM	-2.64	6.16E-04
REACTOME_G1_S_DNA_DAMAGE_CHECKPOINTS	-2.63	6.67E-04
REACTOME_SARS_COV_1_INFECTION	-2.59	8.55E-04
KEGG_MEDICUS_VARIANT_MUTATION_INACTIVATED_VCP_TO_26S_PROTEASOME_MEDIATED_PROTEIN_DEGRADATION	-2.59	8.55E-04
WP_NUCLEOTIDE_EXCISION_REPAIR_IN_XERODERMA_PIGMENTOSUM	-2.59	8.69E-04
REACTOME_SYNTHESIS_OF_DNA	-2.59	8.62E-04

KEGG_MEDICUS_VARIANT_MUTATION_INACTIVATED_UBQLN2_TO_26S_PROTEASOME_MEDIATED_PROTEIN_DEGRADATION	-2.58	9.43E-04
REACTOME_CYCLIN_A_CDK2_ASSOCIATED_EVENTS_AT_S_PHASE_ENTRY	-2.58	9.62E-04
REACTOME_APC_C_CDH1_MEDIATED_DEGRADATION_OF_CDC20_AND_OTHER_APC_C_CDH1_TARGETED_PROTEINS_IN_LATE_MITOSIS_EARLY_G1	-2.56	1.09E-03
PID_BARD1_PATHWAY	-2.55	1.20E-03
REACTOME_RHO_GTPASES_ACTIVATE_FORMINS	-2.54	1.26E-03
REACTOME_DNA_REPLICATION	-2.54	1.29E-03
REACTOME_RNA_POLYMERASE_I_PROMOTER_ESCAPE	-2.53	1.32E-03
REACTOME_DEGRADATION_OF_GLI1_BY_THE_PROTEASOME	-2.52	1.48E-03
REACTOME_TELOMERE_MAINTENANCE	-2.52	1.54E-03
KEGG_TERPENOID_BACKBONE_BIOSYNTHESIS	-2.52	1.53E-03
REACTOME_RUNX1_REGULATES_TRANSCRIPTION_OF_GENES_INVOLVED_IN_DIFFERENTIATION_OF_HSCS	-2.51	1.54E-03
REACTOME_FORMATION_OF_THE_EARLY_ELONGATION_COMPLEX	-2.51	1.54E-03
REACTOME_HDR_THROUGH_SINGLE_STRAND_ANNEALING_SSA	-2.50	1.66E-03
KEGG_MEDICUS_REFERENCE_DNA_END_RESECTION_AND_RPA_LOADING	-2.50	1.66E-03
REACTOME_CLEC7A_DECTIN_1_SIGNALING	-2.50	1.65E-03
REACTOME_PTEN_REGULATION	-2.49	1.68E-03
WP_SREBF_AND_MIR33_IN_CHOLESTEROL_AND_LIPID_HOMEOSTASIS	-2.49	1.69E-03
KEGG_MEDICUS_REFERENCE_MITOCHONDRIAL_COMPLEX_UCP1_IN_THERMOGENESIS	-2.49	1.69E-03
REACTOME_SOMITOGENESIS	-2.48	1.77E-03
WP_REGULATION_OF_SISTER_CHROMATID_SEPARATION_AT_THE_METAPHASE_ANAPHASE_TRANSITION	-2.48	1.79E-03
REACTOME_FORMATION_OF_PARAXIAL_MESODERM	-2.48	1.80E-03
REACTOME_REGULATION_OF_TP53_ACTIVITY_THROUGH_PHOSPHORYLATION	-2.48	1.81E-03
REACTOME_DEFECTIVE_CFTR_CAUSES_CYSTIC_FIBROSIS	-2.47	1.82E-03
REACTOME_ORC1_REMOVAL_FROM_CHROMATIN	-2.47	1.83E-03
KEGG_NON_HOMOLOGOUS_END_JOINING	-2.47	1.83E-03
HALLMARK_INTERFERON_ALPHA_RESPONSE	-2.47	1.86E-03
REACTOME_RNA_POLYMERASE_I_TRANSCRIPTION_TERMINATION	-2.47	1.89E-03
REACTOME_RHO_GTPASE_CYCLE	-2.46	1.89E-03
REACTOME_CILIUM_ASSEMBLY	-2.46	1.98E-03
KEGG_AMINOACYL_TRNA_BIOSYNTHESIS	-2.45	2.07E-03
REACTOME_ROLE_OF_ABL_IN_ROBO_SLIT_SIGNALING	-2.45	2.06E-03
WP_MEVALONATE_PATHWAY	-2.44	2.13E-03

REACTOME_SARS_COV_2_ACTIVATES_MODULATES_INNATE_AND_ADAPTIVE_IMMUNE_RESPONSES	-2.44	2.15E-03
REACTOME_SCF_SKP2_MEDIATED_DEGRADATION_OF_P27_P21	-2.44	2.16E-03
KEGG_MEDICUS_REFERENCE_CHOLESTEROL_BIOSYNTHESIS	-2.43	2.23E-03
WP_MEVALONATE_ARM_OF_CHOLESTEROL_BIOSYNTHESIS_PATHWAY	-2.43	2.35E-03
KEGG_HOMOLOGOUS_RECOMBINATION	-2.43	2.36E-03
WP_CILIOPATHIES	-2.42	2.46E-03
WP_PENTOSE_PHOSPHATE_METABOLISM	-2.42	2.49E-03
REACTOME_APOPTOSIS	-2.42	2.52E-03
REACTOME_POSITIVE_EPIGENETIC_REGULATION_OF_RRNA_EXPRESSION	-2.41	2.60E-03
REACTOME_MITOCHONDRIAL_BIOGENESIS	-2.41	2.63E-03
REACTOME_TRANSCRIPTIONAL_REGULATION_BY_SMALL_RNAS	-2.41	2.62E-03
REACTOME_MRNA_CAPPING	-2.41	2.66E-03
REACTOME_NUCLEAR_ENVELOPE_BREAKDOWN	-2.41	2.65E-03
REACTOME_TRANSCRIPTION_OF_THE_HIV_GENOME	-2.41	2.66E-03
KEGG_MEDICUS_REFERENCE_MEVALONATE_PATHWAY	-2.41	2.65E-03
KEGG_CELL_CYCLE	-2.40	2.85E-03
REACTOME_TRNA_MODIFICATION_IN_THE_NUCLEUS_AND_CYTOSOL	-2.40	2.86E-03
REACTOME_TRANSPORT_OF_MATURE_TRANSCRIPT_TO_CYTOPLASM	-2.39	2.95E-03
REACTOME_RECRUITMENT_OF_MITOTIC_CENTROSOME_PROTEINS_AND_COMPLEXES	-2.39	2.96E-03
BIOCARTA_RANMS_PATHWAY	-2.39	2.99E-03
REACTOME_SNRNP_ASSEMBLY	-2.39	2.98E-03
REACTOME_INTERACTIONS_OF_REV_WITH_HOST_CELLULAR_PROTEINS	-2.38	3.02E-03
REACTOME_MITOTIC_G1_PHASE_AND_G1_S_TRANSITION	-2.38	3.01E-03
WP_PENTOSE_PHOSPHATE_PATHWAY_IN_SENESCENT_CELLS	-2.38	3.06E-03
REACTOME_DNA_REPLICATION_PRE_INITIATION	-2.38	3.10E-03
BIOCARTA_MALATEX_PATHWAY	-2.37	3.31E-03
REACTOME_GENE_SILENCING_BY_RNA	-2.36	3.56E-03
WP_DNA_IR_DOUBLE_STRAND_BREAKS_AND_CELLULAR_RESPONSE_VIA_ATM	-2.35	3.75E-03
REACTOME_HOMOLOGOUS_DNA_PAIRING_AND_STRAND_EXCHANGE	-2.35	3.83E-03
REACTOME_EXTENSION_OF_TELOMERES	-2.35	3.88E-03
REACTOME_MRNA_SPLICING_MINOR_PATHWAY	-2.34	3.99E-03
REACTOME_MITOCHONDRIAL_PROTEIN_IMPORT	-2.34	4.06E-03
REACTOME_REGULATION_OF_RUNX2_EXPRESSION_AND_ACTIVITY	-2.34	4.08E-03

REACTOME_SUMOYLATION_OF_DNA_DAMAGE_RESPONSE_AND_REPAIR_PROTEINS	-2.34	4.09E-03
PID_FANCONI_PATHWAY	-2.33	4.25E-03
PID_TELOMERASE_PATHWAY	-2.33	4.23E-03
REACTOME_REGULATION_OF_RUNX3_EXPRESSION_AND_ACTIVITY	-2.33	4.21E-03
REACTOME_TRNA_PROCESSING_IN_THE_NUCLEUS	-2.33	4.23E-03
REACTOME_PROTEIN_LOCALIZATION	-2.33	4.34E-03
REACTOME_SWITCHING_OF_ORIGINS_TO_A_POST_REPLICATIVE_STATE	-2.32	4.51E-03
REACTOME_PROGRAMMED_CELL_DEATH	-2.32	4.56E-03
REACTOME_VIRAL_MESSENGER_RNA_SYNTHESIS	-2.31	4.71E-03
REACTOME_PROCESSING_OF_DNA_DOUBLE_STRAND_BREAK_ENDS	-2.31	4.96E-03
REACTOME_PENTOSE_PHOSPHATE_PATHWAY	-2.29	5.34E-03
REACTOME_HIV_TRANSCRIPTION_INITIATION	-2.29	5.33E-03
REACTOME_RECRUITMENT_OF_NUMA_TO_MITOTIC_CENTROSOMES	-2.29	5.61E-03
REACTOME_REGULATION_OF_PLK1_ACTIVITY_AT_G2_M_TRANSITION	-2.29	5.59E-03
REACTOME_HDR_THROUGH_MMEJ_ALT_NHEJ	-2.28	5.62E-03
REACTOME_DUAL_INCISION_IN_TC_NER	-2.28	6.02E-03
WP_CELL_CYCLE	-2.27	6.23E-03
REACTOME_CRISTAE_FORMATION	-2.27	6.25E-03
REACTOME_HIV_TRANSCRIPTION_ELONGATION	-2.27	6.25E-03
HALLMARK_INTERFERON_GAMMA_RESPONSE	-2.26	6.60E-03
REACTOME_DISEASES_OF_DNA_REPAIR	-2.26	6.61E-03
KEGG_MEDICUS_REFERENCE_ELECTRON_TRANSFER_IN_COMPLEX_IV	-2.26	6.63E-03
REACTOME_RHOBTB1_GTPASE_CYCLE	-2.26	6.61E-03
REACTOME_HATS_ACETYLATE_HISTONES	-2.26	6.66E-03
REACTOME_RHOBTB2_GTPASE_CYCLE	-2.25	6.74E-03
REACTOME_HDR_THROUGH_HOMOLOGOUS_RECOMBINATION_HRR	-2.25	6.84E-03
REACTOME_RNA_POLYMERASE_II_TRANSCRIPTION_TERMINATION	-2.25	6.94E-03
REACTOME_ASSOCIATION_OF_TRIC_CCT_WITH_TARGET_PROTEINS_DURING_BIOSYNTHESIS	-2.24	7.27E-03
REACTOME_BASE_EXCISION_REPAIR	-2.24	7.36E-03
REACTOME_RESOLUTION_OF_D_LOOP_STRUCTURES	-2.23	7.39E-03
WP_MITOCHONDRIAL_COMPLEX_IV_ASSEMBLY	-2.23	7.55E-03
BIOCARTA_HSWI_SNF_PATHWAY	-2.23	7.54E-03
WP_EUKARYOTIC_TRANSCRIPTION_INITIATION	-2.23	7.51E-03
REACTOME_TELOMERE_C_STRAND_LAGGING_STRAND_SYNTHESIS	-2.23	7.61E-03
REACTOME_ASYMMETRIC_LOCALIZATION_OF_PCP_PROTEINS	-2.23	7.58E-03

KEGG_VALINE_LEUCINE_AND_ISOLEUCINE_BIOSYNTHESIS	-2.23	7.55E-03
PID_AR_PATHWAY	-2.23	7.59E-03
WP_SMC1_SMC3_ROLE_IN_DNA_DAMAGE_CORNELIA_DE_LANGE_SYNDROME	-2.22	7.77E-03
KEGG_PORPHYRIN_AND_CHLOROPHYLL_METABOLISM	-2.22	7.92E-03
REACTOME_ANCHORING_OF_THE_BASAL_BODY_TO_THE_PLASMA_MEMBRANE	-2.22	8.06E-03
KEGG_MEDICUS_VARIANT_MUTATION_CAUSED_ABERRANT_ABETA_TO_ELECTRON_TRANSFER_IN_COMPLEX_IV	-2.21	8.30E-03
BIOCARTA_ATRBRCA_PATHWAY	-2.20	8.65E-03
REACTOME_EPIGENETIC_REGULATION_OF_GENE_EXPRESSION	-2.20	9.03E-03
REACTOME_SIGNALING_BY_FGFR2_IIIA_TM	-2.20	9.06E-03
PID_DNA_PK_PATHWAY	-2.20	9.06E-03
WP_CHOLESTEROL_METABOLISM	-2.18	1.03E-02
WP_METABOLIC_REPROGRAMMING_IN_COLON_CANCER	-2.17	1.10E-02
REACTOME_TRANSCRIPTIONAL_REGULATION_BY_RUNX1	-2.16	1.13E-02
WP_METABOLIC_REPROGRAMMING_IN_PANCREATIC_CANCER	-2.16	1.16E-02
REACTOME_NS1_MEDIATED_EFFECTS_ON_HOST_PATHWAYS	-2.16	1.17E-02
WP_NON_HOMOLOGOUS_END_JOINING	-2.14	1.25E-02
REACTOME_ESTROGEN_DEPENDENT_NUCLEAR_EVENTS_DOWNSTREAM_OF_ESR_MEMBRANE_SIGNALING	-2.14	1.28E-02
KEGG_MEDICUS_ENV_FACTOR_ARSENIC_TO_ELECTRON_TRANSFER_IN_COMPLEX_IV	-2.14	1.31E-02
REACTOME_TRANSPORT_OF_MATURE_MRNAS_DERIVED_FROM_INTRONLESS_TRANSCRIPTS	-2.13	1.33E-02
KEGG_MEDICUS_REFERENCE_NUCLEAR_EXPORT_OF_MRNA	-2.13	1.36E-02
WP_ATM_SIGNALING_IN_DEVELOPMENT_AND_DISEASE	-2.12	1.41E-02
HALLMARK_ANDROGEN_RESPONSE	-2.12	1.41E-02
REACTOME_B_WICH_COMPLEX_POSITIVELY_REGULATES_RRNA_EXPRESSION	-2.12	1.41E-02
REACTOME_DOWNSTREAM_SIGNALING_EVENTS_OF_B_CELL_RECEPTOR_BCR	-2.12	1.41E-02
WP_TCA_CYCLE_IN_SENESCENCE	-2.12	1.43E-02
REACTOME_RNA_POLYMERASE_III_TRANSCRIPTION	-2.11	1.48E-02
REACTOME_TCR_SIGNALING	-2.10	1.56E-02
REACTOME_DEPOLYMERIZATION_OF_THE_NUCLEAR_LAMINA	-2.10	1.59E-02
WP_UREA_CYCLE_AND_ASSOCIATED_PATHWAYS	-2.10	1.58E-02
WP_RETINOBLASTOMA_GENE_IN_CANCER	-2.10	1.62E-02
KEGG_MEDICUS_REFERENCE_RECRUITMENT_AND_FORMATION_OF_THE_MCC	-2.09	1.70E-02
WP_PROTEASOME_DEGRADATION	-2.08	1.78E-02
REACTOME_NUCLEAR_IMPORT_OF_REV_PROTEIN	-2.08	1.77E-02
HALLMARK_UNFOLDED_PROTEIN_RESPONSE	-2.08	1.81E-02

REACTOME_POLB_DEPENDENT_LONG_PATCH_BASE_EXCISION_REPAIR	-2.07	1.87E-02
REACTOME_THE_ROLE_OF_GTSE1_IN_G2_M_PROGRESSION_AFTER_G2_CHECKPOINT	-2.07	1.89E-02
KEGG_MEDICUS_REFERENCE_NON_HOMOLOGOUS_END_JOINING	-2.07	1.91E-02
WP_FERROPTOSIS	-2.06	2.07E-02
BIOCARTA_BARD1_PATHWAY	-2.05	2.08E-02
REACTOME_AURKA_ACTIVATION_BY_TPX2	-2.05	2.10E-02
REACTOME_SIGNALING_BY_NOTCH4	-2.05	2.15E-02
REACTOME_RESOLUTION_OF_ABASIC_SITES_AP_SITES	-2.04	2.24E-02
REACTOME_DEADENYLATION_DEPENDENT_MRNA_DECAY	-2.03	2.31E-02
REACTOME_CHROMATIN_MODIFYING_ENZYMES	-2.03	2.43E-02
REACTOME_SYNTHESIS_OF_DIPHTHAMIDE_EEF2	-2.03	2.44E-02
REACTOME_NEGATIVE_EPIGENETIC_REGULATION_OF_RRNA_EXPRESSION	-2.02	2.46E-02
REACTOME_CELLULAR_RESPONSE_TO_HYPOXIA	-2.02	2.48E-02
REACTOME_TP53_REGULATES_METABOLIC_GENES	-2.02	2.48E-02
REACTOME_SUMOYLATION_OF_DNA_REPLICATION_PROTEINS	-2.02	2.47E-02
REACTOME_NFE2L2_REGULATING_ANTI_OXIDANT_DETOXIFICATION_ENZYMES	-2.02	2.48E-02
KEGG_PYRUVATE_METABOLISM	-2.02	2.54E-02
KEGG_MEDICUS_REFERENCE_HOMOLOGOUS_RECOMBINATION_IN_ICLR	-2.01	2.57E-02
KEGG_MEDICUS_REFERENCE_INACTIVATION_OF_CONDENSIN_I	-2.01	2.61E-02
WP_BASE_EXCISION_REPAIR	-2.01	2.65E-02
REACTOME_METABOLISM_OF_STEROIDS	-2.01	2.64E-02
WP_PRIMARY_OVARIAN_INSUFFICIENCY	-2.01	2.63E-02
REACTOME_ABORTIVE_ELONGATION_OF_HIV_1_TRANSCRIPT_IN_THE_ABSENCE_OF_TAT	-2.00	2.71E-02
KEGG_MEDICUS_REFERENCE_DEPHOSPHORYLATION_OF_KINETOCHORE	-2.00	2.74E-02
WP_MITOCHONDRIAL_COMPLEX_III_ASSEMBLY	-1.99	2.86E-02
WP_MITOCHONDRIAL_COMPLEX_I_ASSEMBLY_MODEL_OXPHOS_SYSTEM	-1.99	2.85E-02
REACTOME_RNA_POLYMERASE_III_TRANSCRIPTION_INITIATION_FROM_TYPE_3_PROMOTER	-1.99	2.85E-02
HALLMARK_REACTIVE_OXYGEN_SPECIES_PATHWAY	-1.99	2.89E-02
REACTOME_RESOLUTION_OF_D_LOOP_STRUCTURES_THROUGH_SYNTHESIS_DEPENDENT_STRAND_ANNEALING_SDSA	-1.99	2.92E-02
REACTOME_INTERACTIONS_OF_VPR_WITH_HOST_CELLULAR_PROTEINS	-1.99	2.92E-02
REACTOME_ABC_FAMILY_PROTEINS_MEDIATED_TRANSPORT	-1.98	3.02E-02
REACTOME_NEDDYLATION	-1.98	3.02E-02

WP_TCA_CYCLE_AND_DEFICIENCY_OF_PYRUVATE_DEHYDROGENASE_COMPLEX_PDHC	-1.98	3.06E-02
REACTOME_REGULATION_OF_RAS_BY_GAPS	-1.97	3.17E-02
KEGG_CITRATE_CYCLE_TCA_CYCLE	-1.97	3.24E-02
REACTOME_TIGHT_JUNCTION_INTERACTIONS	-1.96	3.30E-02
BIOCARTA_ETC_PATHWAY	-1.96	3.33E-02
REACTOME_CONDENSATION_OF_PROMETAPHASE_CHROMOSOMES	-1.95	3.48E-02
REACTOME_RNA_POLYMERASE_III_TRANSCRIPTION_TERMINATION	-1.95	3.49E-02
WP_GASTRIC_CANCER_NETWORK_2	-1.95	3.52E-02
WP_LIVER_X_RECEPTOR_PATHWAY	-1.95	3.56E-02
REACTOME_METABOLISM_OF_PORPHYRINS	-1.94	3.61E-02
REACTOME_EXPORT_OF_VIRAL_RIBONUCLEOPROTEINS_FROM_NUCLEUS	-1.94	3.70E-02
REACTOME_BBSOME_MEDIATED_CARGO_TARGETING_TO_CILIUM	-1.93	3.90E-02
REACTOME_NUCLEOTIDE_BIOSYNTHESIS	-1.92	4.05E-02
KEGG_PYRIMIDINE_METABOLISM	-1.91	4.32E-02
REACTOME_RNA_POLYMERASE_II_PRE_TRANSCRIPTION_EVENTS	-1.91	4.32E-02
BIOCARTA_ERBB3_PATHWAY	-1.91	4.38E-02
REACTOME_PROCESSIVE_SYNTHESIS_ON_THE_C_STRAND_OF_THE_TELOMERE	-1.91	4.37E-02
REACTOME_UPTAKE_AND_FUNCTION_OF_DIPHTHERIA_TOXIN	-1.91	4.37E-02
BIOCARTA_PROTEASOME_PATHWAY	-1.91	4.38E-02
REACTOME_TNFR2_NON_CANONICAL_NF_KB_PATHWAY	-1.91	4.40E-02
REACTOME_DNA_DAMAGE_RECOGNITION_IN_GG_NER	-1.90	4.40E-02
REACTOME_REGULATION_OF_TP53_ACTIVITY_THROUGH_ASSOCIATION_WITH_CO_FACTORS	-1.90	4.46E-02
WP_NSP1_FROM_SARS_COV_2_INHIBITS_TRANSLATION_INITIATION_IN_THE_HOST_CELL	-1.90	4.46E-02
WP_7Q11_23_COPY_NUMBER_VARIATION_SYNDROME	-1.90	4.45E-02
REACTOME_ABC_TRANSPORTER_DISORDERS	-1.90	4.45E-02
REACTOME_OAS_ANTIVIRAL_RESPONSE	-1.90	4.50E-02
REACTOME_FORMATION_OF_WDR5_CONTAINING_HISTONE_MODIFYING_COMPLEXES	-1.89	4.59E-02
REACTOME_APOPTOTIC_CLEAVAGE_OF_CELL_ADHESION_PROTEINS	-1.89	4.75E-02
WP_CILIARY_LANDSCAPE	-1.89	4.75E-02
PID_MYC_PATHWAY	-1.89	4.76E-02
KEGG_RNA_DEGRADATION	-1.89	4.76E-02
REACTOME_FORMATION_OF_INCISION_COMPLEX_IN_GG_NER	-1.88	4.95E-02
WP_BILE_ACIDS_SYNTHESIS_AND_ENTEROHEPATIC_CIRCULATION	-1.88	4.96E-02

Supplementary Table S2. List of differentially altered gene sets in *CERS5/6* KD cardiomyocytes (FDR < 0.05).

Gene Set Name	Normalized Enrichment Score (NES)	FDR
HALLMARK_INTERFERON_ALPHA_RESPONSE	7.60	0.00E+00
REACTOME_TRANSLATION	6.95	0.00E+00
HALLMARK_INTERFERON_GAMMA_RESPONSE	6.89	0.00E+00
REACTOME_RRNA_PROCESSING	5.75	0.00E+00
HALLMARK_OXIDATIVE_PHOSPHORYLATION	5.70	0.00E+00
REACTOME_EUKARYOTIC_TRANSLATION_INITIATION	5.59	0.00E+00
KEGG_MEDICUS_REFERENCE_TRANSLATION_INITIATION	5.27	0.00E+00
WP_CYTOPLASMIC_RIBOSOMAL_PROTEINS	5.15	0.00E+00
REACTOME_SELENOAMINO_ACID_METABOLISM	5.05	0.00E+00
REACTOME_EUKARYOTIC_TRANSLATION_ELONGATION	4.99	0.00E+00
KEGG_RIBOSOME	4.95	0.00E+00
REACTOME_INTERFERON_ALPHA_BETA_SIGNALING	4.89	0.00E+00
REACTOME_SRP_DEPENDENT_COTRANSLATIONAL_PROTEIN_TARGETING_TO_MEMBRANE	4.87	0.00E+00
HALLMARK_TNFA_SIGNALING_VIA_NFKB	4.80	0.00E+00
REACTOME_RESPONSE_OF_EIF2AK4_GCN2_TO_AMINO_ACID_DEFICIENCY	4.64	0.00E+00
REACTOME_NONSENSE_MEDIATED_DECAY_NMD	4.62	0.00E+00
REACTOME_CELLULAR_RESPONSE_TO_STARVATION	4.54	0.00E+00
REACTOME_ACTIVATION_OF_THE_MRNA_UPON_BINDING_OF_THE_CAP_BINDING_COMPLEX_AND_EIFS_AND_SUBSEQUENT_BINDING_TO_43S	4.50	0.00E+00
REACTOME_MITOCHONDRIAL_TRANSLATION	4.45	0.00E+00
REACTOME_REGULATION_OF_EXPRESSION_OF_SLITS_AND_ROBOS	4.38	0.00E+00
REACTOME_THE_CITRIC_ACID_TCA_CYCLE_AND_RESPIRATORY_ELECTRON_TRANSPORT	4.31	0.00E+00
REACTOME_RESPIRATORY_ELECTRON_TRANSPORT_ATP_SYNTHESIS_BY_CHEMIOSMOTIC_COUPLING_AND_HEAT_PRODUCTION_BY_UNCOUPLING_PROTEINS	4.20	0.00E+00
HALLMARK_P53_PATHWAY	4.15	0.00E+00
WP_ELECTRON_TRANSPORT_CHAIN_OXPHOS_SYSTEM_IN_MITOCHONDRIA	4.12	0.00E+00
KEGG_PARKINSONS_DISEASE	4.11	0.00E+00
REACTOME_RESPIRATORY_ELECTRON_TRANSPORT	4.08	0.00E+00
REACTOME_METABOLISM_OF_AMINO_ACIDS_AND_DERIVATIVES	4.03	0.00E+00
KEGG_OXIDATIVE_PHOSPHORYLATION	4.02	0.00E+00
REACTOME_INFLUENZA_INFECTION	4.02	0.00E+00

REACTOME_SARS_COV_1_MODULATES_HOST_TRANSLATION_MACHINERY	4.02	0.00E+00
REACTOME_SARS_COV_2_MODULATES_HOST_TRANSLATION_MACHINERY	3.87	0.00E+00
KEGG_PURINE_METABOLISM	3.79	0.00E+00
REACTOME_INTERFERON_SIGNALING	3.78	0.00E+00
KEGG_MEDICUS_PATHOGEN_SARS_COV_2_NSP1_TO_TRANSLATION_INITIATION	3.76	0.00E+00
KEGG_ALZHEIMERS_DISEASE	3.72	0.00E+00
WP_VEGFA_VEGFR2_SIGNALING	3.68	0.00E+00
REACTOME_SIGNALING_BY_ROBO_RECEPTORS	3.64	0.00E+00
HALLMARK_DNA_REPAIR	3.49	0.00E+00
KEGG_PYRIMIDINE_METABOLISM	3.42	0.00E+00
KEGG_MEDICUS_REFERENCE_MITOCHONDRIAL_COMPLEX_UCP1_IN_THERMOGENESIS	3.37	0.00E+00
REACTOME_SARS_COV_1_INFECTION	3.34	0.00E+00
HALLMARK_MYC_TARGETS_V1	3.29	0.00E+00
KEGG_HUNTINGTONS_DISEASE	3.23	0.00E+00
HALLMARK_MYC_TARGETS_V2	3.19	0.00E+00
KEGG_MEDICUS_VARIANT_MUTATION_CAUSED_ABERRANT_SNCA_TO_ELECTRON_TRANSFER_IN_COMPLEX_I	3.18	0.00E+00
KEGG_MEDICUS_VARIANT_MUTATION_INACTIVATED_PINK1_TO_ELECTRON_TRANSFER_IN_COMPLEX_I	3.17	0.00E+00
REACTOME_SARS_COV_1_HOST_INTERACTIONS	3.13	0.00E+00
KEGG_MEDICUS_VARIANT_MUTATION_CAUSED_ABERRANT_ABETA_TO_ELECTRON_TRANSFER_IN_COMPLEX_I	3.11	0.00E+00
WP_OXIDATIVE_PHOSPHORYLATION	3.08	4.50E-05
KEGG_MEDICUS_REFERENCE_ELECTRON_TRANSFER_IN_COMPLEX_I	3.08	4.41E-05
WP_SARS_COV_2_INNATE_IMMUNITY_EVASION_AND_CELL_SPECIFIC_IMMUNE_RESPONSE	3.06	6.52E-05
WP_NONALCOHOLIC_FATTY_LIVER_DISEASE	3.05	6.40E-05
HALLMARK_ADIPOGENESIS	3.02	8.33E-05
WP_CYTOSOLIC_DNA_SENSING_PATHWAY	3.01	1.03E-04
WP_MEASLES_VIRUS_INFECTION	3.01	1.01E-04
REACTOME_INTERFERON_GAMMA_SIGNALING	2.96	1.19E-04
HALLMARK_E2F_TARGETS	2.95	1.17E-04
REACTOME_MITOCHONDRIAL_PROTEIN_IMPORT	2.94	1.15E-04
KEGG_CYTOSOLIC_DNA_SENSING_PATHWAY	2.93	1.32E-04
KEGG_MEDICUS_VARIANT_MUTATION_CAUSED_ABERRANT_TDP43_TO_ELECTRON_TRANSFER_IN_COMPLEX_I	2.89	1.84E-04
REACTOME_PROTEIN_LOCALIZATION	2.89	1.81E-04
REACTOME_COMPLEX_I_BIOGENESIS	2.89	1.78E-04

WP_TYPE_II_INTERFERON_SIGNALING	2.87	1.75E-04
REACTOME_SIGNALING_BY_INTERLEUKINS	2.86	2.08E-04
REACTOME_RRNA_MODIFICATION_IN_THE_NUCLEUS_AND_CYTOSOL	2.84	3.24E-04
WP_P53_TRANSCRIPTIONAL_GENE_NETWORK	2.80	3.86E-04
REACTOME_CYTOSOLIC_SENSORS_OF_PATHOGEN_ASSOCIATED_DNA	2.80	3.80E-04
KEGG_MEDICUS_PATHOGEN_HTLV_1_P12_TO_ANTIGEN_PROCESSING_AND_PRESENTATION_BY_MHC_CLASS_I_MOLECULES	2.79	4.23E-04
REACTOME_TRANSCRIPTION_COUPLED_NUCLEOTIDE_EXCISION_REPAIR_TC_NER	2.77	5.45E-04
REACTOME_NUCLEOTIDE_EXCISION_REPAIR	2.76	5.53E-04
KEGG_P53_SIGNALING_PATHWAY	2.75	6.39E-04
REACTOME_SARS_COV_2_HOST_INTERACTIONS	2.75	6.46E-04
KEGG_MEDICUS_REFERENCE_MGLUR5_CA2_APOPTOTIC_PATHWAY	2.74	6.67E-04
KEGG_MEDICUS_VARIANT_MUTATION_CAUSED_ABERRANT_ABETA_TO_MGLUR5_CA2_APOPTOTIC_PATHWAY	2.73	6.73E-04
HALLMARK_UV_RESPONSE_UP	2.71	7.37E-04
WP_DNA_REPLICATION	2.70	7.86E-04
KEGG_MEDICUS_REFERENCE_INTRINSIC_APOPTOTIC_PATHWAY	2.68	8.19E-04
REACTOME_PYROPTOSIS	2.68	8.65E-04
KEGG_MEDICUS_PATHOGEN_KSHV_MIR1_2_TO_ANTIGEN_PROCESSING_AND_PRESENTATION_BY_MHC_CLASS_I_MOLECULES	2.67	8.96E-04
KEGG_CHEMOKINE_SIGNALING_PATHWAY	2.66	9.13E-04
PID_PDGFRB_PATHWAY	2.66	9.02E-04
WP_APOPTOSIS_MODULATION_AND_SIGNALING	2.66	9.04E-04
WP_17Q12_COPY_NUMBER_VARIATION_SYNDROME	2.66	9.46E-04
PID_P53_DOWNSTREAM_PATHWAY	2.65	9.48E-04
REACTOME_PYRUVATE_METABOLISM_AND_CITRIC_ACID_TCA_CYCLE	2.62	1.22E-03
WP_NUCLEOTIDE_EXCISION_REPAIR_IN_XERODERMA_PIGMENTOSUM	2.62	1.26E-03
REACTOME_SARS_COV_INFECTIONS	2.61	1.31E-03
WP_THYMIC_STROMAL_LYMPHOPOIETIN_TSLP_SIGNALING_PATHWAY	2.61	1.34E-03
REACTOME_NEUTROPHIL_DEGRANULATION	2.59	1.40E-03
WP_MITOCHONDRIAL_COMPLEX_I_ASSEMBLY_MODEL_OXPHOS_SYSTEM	2.58	1.46E-03
REACTOME_HIV_TRANSCRIPTION_ELONGATION	2.57	1.63E-03
WP_NETWORK_MAP_OF_SARS_COV_2_SIGNALING_PATHWAY	2.57	1.77E-03
REACTOME_SARS_COV_2_INFECTION	2.56	1.81E-03
REACTOME_ENDOSOMAL_VACUOLAR_PATHWAY	2.55	1.94E-03
WP_IMMUNE_RESPONSE_TO_TUBERCULOSIS	2.55	1.99E-03

WP_INTERLEUKIN_1_IL_1_STRUCTURAL_PATHWAY	2.55	1.98E-03
REACTOME_ACTIVATION_OF_THE_PRE_REPLICATIVE_COMPLEX	2.55	1.98E-03
WP_NON_SMALL_CELL_LUNG_CANCER	2.54	2.04E-03
KEGG_MEDICUS_REFERENCE_ANTIGEN_PROCESSING_AND_PRESENTATION_BY_MHC_CLASS_I_MOLECULES	2.53	2.27E-03
HALLMARK_ESTROGEN_RESPONSE_LATE	2.53	2.24E-03
WP_GASTRIN_SIGNALING_PATHWAY	2.53	2.24E-03
REACTOME_RNA_POLYMERASE_III_CHAIN_ELONGATION	2.52	2.23E-03
KEGG_MEDICUS_VARIANT_MUTATION_CAUSED_ABERRANT_SNCA_TO_VGCC_CA2_APOPTOTIC_PATHWAY	2.52	2.27E-03
KEGG_DNA_REPLICATION	2.50	2.73E-03
REACTOME_DNA_REPAIR	2.50	2.73E-03
REACTOME_RIBAVIRIN_ADME	2.49	2.83E-03
KEGG_MEDICUS_VARIANT_MUTATION_CAUSED_ABERRANT_HTT_TO_MGLUR5_CA2_APOPTOTIC_PATHWAY	2.49	2.87E-03
REACTOME_RNA_POLYMERASE_III_TRANSCRIPTION_INITIATION_FROM_TYPE_1_PROMOTER	2.49	2.87E-03
WP_EUKARYOTIC_TRANSCRIPTION_INITIATION	2.47	3.08E-03
WP_EBOLA_VIRUS_INFECTION_IN_HOST	2.47	3.17E-03
KEGG_MEDICUS_VARIANT_MUTATION_CAUSED_ABERRANT_PSEN_TO_MGLUR5_CA2_APOPTOTIC_PATHWAY	2.47	3.25E-03
REACTOME_METABOLISM_OF_NUCLEOTIDES	2.46	3.27E-03
KEGG_BUTANOATE_METABOLISM	2.46	3.25E-03
WP_CHEMOKINE_SIGNALING_PATHWAY	2.45	3.71E-03
REACTOME_MITOCHONDRIAL_CALCIUM_ION_TRANSPORT	2.44	3.77E-03
REACTOME_GLOBAL_GENOME_NUCLEOTIDE_EXCISION_REPAIR_GG_NER	2.40	5.36E-03
REACTOME_SIGNALING_BY_NTRKS	2.39	5.62E-03
KEGG_MEDICUS_VARIANT_MUTATION_CAUSED_ABERRANT_ABETA_TO_MACHR_CA2_APOPTOTIC_PATHWAY	2.38	5.85E-03
WP_OVERVIEW_OF_PROINFLAMMATORY_AND_PROFIBROTIC_MEDIATORS	2.38	6.10E-03
REACTOME_TOLL_LIKE_RECEPTOR_CASCADES	2.37	6.26E-03
WP_PRE_IMPLANTATION_EMBRYO	2.37	6.37E-03
REACTOME_HIV_ELONGATION_ARREST_AND_RECOVERY	2.36	6.71E-03
KEGG_MEDICUS_ENV_FACTOR_ARSENIC_TO_ELECTRON_TRANSFER_IN_COMPLEX_IV	2.35	7.45E-03
KEGG_NEUROTROPHIN_SIGNALING_PATHWAY	2.32	9.16E-03
WP_NUCLEOTIDE_EXCISION_REPAIR	2.32	9.18E-03
KEGG_MEDICUS_REFERENCE_ORIGIN_UNWINDING_AND_ELONGATION	2.32	9.31E-03
REACTOME_DUAL_INCISION_IN_TC_NER	2.32	9.25E-03

KEGG_MEDICUS_PATHOGEN_HPV_E5_TO_ANTIGEN_PROCESSING_AND_PRESENTATION_BY_MHC_CLASS_I_MOLECULES	2.31	9.30E-03
REACTOME_FORMATION_OF_RNA_POL_II_ELONGATION_COMPLEX	2.31	9.46E-03
REACTOME_FORMATION_OF_TC_NER_PRE_INCISION_COMPLEX	2.30	9.92E-03
REACTOME_MYD88_INDEPENDENT_TLR4_CASCADE	2.30	9.89E-03
HALLMARK_FATTY_ACID_METABOLISM	2.30	9.84E-03
REACTOME_MITOTIC_G1_PHASE_AND_G1_S_TRANSITION	2.30	9.82E-03
PID_TAP63_PATHWAY	2.30	9.97E-03
REACTOME_CITRIC_ACID_CYCLE_TCA_CYCLE	2.29	1.02E-02
REACTOME_METABOLISM_OF_WATER_SOLUBLE_VITAMINS_AND_COFACTORS	2.29	1.06E-02
PID_IL23_PATHWAY	2.29	1.06E-02
PID_IL27_PATHWAY	2.27	1.16E-02
WP_HOST_PATHOGEN_INTERACTION_OF_HUMAN_CORONAVIRUSES_INTERFERON_INDUCTION	2.27	1.17E-02
BIOCARTA_MHC_PATHWAY	2.27	1.21E-02
KEGG_MEDICUS_VARIANT_MUTATION_CAUSED_ABERRANT_ABETA_TO_ELECTRON_TRANSFER_IN_COMPLEX_IV	2.27	1.21E-02
KEGG_MEDICUS_REFERENCE_MACHR_CA2_APOPTOTIC_PATHWAY	2.26	1.22E-02
WP_TP53_NETWORK	2.26	1.22E-02
WP_NSP1_FROM_SARS_COV_2_INHIBITS_TRANSLATION_INITIATION_IN_THE_HOST_CELL	2.26	1.25E-02
KEGG_GLUTATHIONE_METABOLISM	2.25	1.34E-02
WP_TRANSLATION_FACTORS	2.24	1.40E-02
REACTOME_FORMATION_OF_THE_EARLY_ELONGATION_COMPLEX	2.23	1.43E-02
REACTOME_REGULATED_NECROSIS	2.23	1.46E-02
WP_IL_2_SIGNALING_PATHWAY	2.23	1.48E-02
WP_RETINOBLASTOMA_GENE_IN_CANCER	2.23	1.47E-02
KEGG_NUCLEOTIDE_EXCISION_REPAIR	2.23	1.47E-02
KEGG_PYRUVATE_METABOLISM	2.22	1.49E-02
HALLMARK_ESTROGEN_RESPONSE_EARLY	2.22	1.55E-02
REACTOME_TRANSCRIPTIONAL_REGULATION_BY_TP53	2.22	1.56E-02
WP_TCA_CYCLE_AKA_KREBS_OR_CITRIC_ACID_CYCLE	2.21	1.61E-02
REACTOME_RNA_POLYMERASE_III_TRANSCRIPTION_INITIATION_FROM_TYPE_3_PROMOTER	2.21	1.61E-02
KEGG_GALACTOSE_METABOLISM	2.21	1.64E-02
KEGG_VALINE_LEUCINE_AND_ISOLEUCINE_DEGRADATION	2.21	1.65E-02
KEGG_MEDICUS_REFERENCE_ELECTRON_TRANSFER_IN_COMPLEX_IV	2.20	1.75E-02
WP_INCLUSION_BODY_MYOSITIS	2.20	1.74E-02
REACTOME_FGFR1C_LIGAND_BINDING_AND_ACTIVATION	2.19	1.75E-02
KEGG_MEDICUS_VARIANT_MUTATION_INACTIVATED_PINK1_TO_INTRINSIC_APOPTOTIC_PATHWAY_N01048	2.19	1.76E-02

WP_DNA_DAMAGE_RESPONSE	2.19	1.80E-02
PID_IL12_2PATHWAY	2.19	1.80E-02
WP_TOLL_LIKE_RECEPTOR_SIGNALING_PATHWAY	2.18	1.84E-02
REACTOME_PROGRAMMED_CELL_DEATH	2.18	1.85E-02
KEGG_MEDICUS_VARIANT_MUTATION_CAUSED_ABERRANT_ABETA_TO_TRANSPORT_OF_CALCIUM	2.18	1.92E-02
KEGG_MEDICUS_REFERENCE_PRE_IC_FORMATION	2.18	1.91E-02
REACTOME_HIV_INFECTION	2.17	1.96E-02
WP_MIRNA_REGULATION_OF_DNA_DAMAGE_RESPONSE	2.17	1.97E-02
REACTOME_THE_NLRP3_INFLAMMASOME	2.17	1.96E-02
REACTOME_CRISTAE_FORMATION	2.17	1.96E-02
REACTOME_TRANSCRIPTION_OF_THE_HIV_GENOME	2.17	1.99E-02
WP_APOPTOSIS	2.16	2.00E-02
WP_TYPE_I_INTERFERON_INDUCTION_AND_SIGNALING_DURING_SARS_COV_2_INFECTION	2.16	2.08E-02
REACTOME_RNA_POLYMERASE_II_PRE_TRANSCRIPTION_EVENTS	2.16	2.08E-02
KEGG_LYSOSOME	2.16	2.07E-02
PID_REG_GR_PATHWAY	2.15	2.06E-02
REACTOME_NUCLEOTIDE_BINDING_DOMAIN_LEUCINE_RICH_REPEAT_CONTAINING_RECEPTOR_NLR_SIGNALING_PATHWAYS	2.15	2.06E-02
BIOCARTA_CHEMICAL_PATHWAY	2.15	2.06E-02
KEGG_MEDICUS_VARIANT_MUTATION_CAUSED_ABERRANT_PSEN1_TO_MGLUR5_CA2_APOPTOTIC_PATHWAY	2.15	2.06E-02
REACTOME_TOLL_LIKE_RECEPTOR_9_TLR9_CASCADE	2.15	2.06E-02
HALLMARK_INFLAMMATORY_RESPONSE	2.15	2.08E-02
WP_DNA_MISMATCH_REPAIR	2.15	2.08E-02
REACTOME_TP53_REGULATES_TRANSCRIPTION_OF_DNA_REPAIR_GENES	2.15	2.07E-02
WP_MITOCHONDRIAL_COMPLEX_III_ASSEMBLY	2.15	2.07E-02
KEGG_MEDICUS_VARIANT_MUTATION_CAUSED_ABBERANT_ATXN2_3_TO_MGLUR5_CA2_APOPTOTIC_PATHWAY	2.15	2.09E-02
KEGG_MEDICUS_REFERENCE_IL12_23_TO_JAK_STAT_SIGNALING_PATHWAY	2.14	2.09E-02
HALLMARK_COMPLEMENT	2.14	2.13E-02
REACTOME_GLUTATHIONE_CONJUGATION	2.14	2.18E-02
WP_NON_GENOMIC_ACTIONS_OF_1_25_DIHYDROXYVITAMIN_D3	2.14	2.17E-02
REACTOME_TOLL_LIKE_RECEPTOR_TLR1_TLR2_CASCADE	2.13	2.24E-02
WP_T_CELL_MODULATION_IN_PANCREATIC_CANCER	2.13	2.24E-02
WP_STING_PATHWAY_IN_KAWASAKI_LIKE_DISEASE_AND_COVID_19	2.12	2.31E-02
REACTOME_TAK1_DEPENDENT_IKK_AND_NF_KAPPA_B_ACTIVATION	2.12	2.36E-02
PID_LYSOPHOSPHOLIPID_PATHWAY	2.12	2.41E-02
WP_CELLULAR_PROTEOSTASIS	2.11	2.43E-02

HALLMARK_APICAL_JUNCTION	2.11	2.45E-02
WP_KIT_RECEPTOR_SIGNALING_PATHWAY	2.11	2.51E-02
HALLMARK_G2M_CHECKPOINT	2.09	2.80E-02
REACTOME_PURINERGIC_SIGNALING_IN_LEISHMANIASIS_INFECTION	2.09	2.84E-02
KEGG_CARDIAC_MUSCLE_CONTRACTION	2.08	2.91E-02
WP_MYOMETRIAL_RELAXATION_AND_CONTRACTION_PATHWAYS	2.08	2.94E-02
REACTOME_DISEASES_OF_METABOLISM	2.08	2.93E-02
KEGG_MEDICUS_VARIANT_MUTATION_CAUSED_ABERRANT_ABETA_TO_VGCC_CA2_APOPTOTIC_PATHWAY_N01004	2.08	2.95E-02
WP_TNF_ALPHA_SIGNALING_PATHWAY	2.08	2.95E-02
REACTOME_FORMATION_OF_ATP_BY_CHEMIOSMOTIC_COUPLING	2.08	2.95E-02
REACTOME_RELEASE_OF_APOPTOTIC_FACTORS_FROM_THE_MITOCHONDRIA	2.08	2.94E-02
WP_CORTICOTROPIN_RELEASING_HORMONE_SIGNALING_PATHWAY	2.07	2.97E-02
WP_EGFR_TYROSINE_KINASE_INHIBITOR_RESISTANCE	2.07	3.04E-02
WP_MITOCHONDRIAL_COMPLEX_IV_ASSEMBLY	2.07	3.08E-02
KEGG_MEDICUS_VARIANT_SCRAPIE_CONFORMATION_PRPSC_TO_TRANSPORT_OF_CALCIUM	2.07	3.08E-02
REACTOME_INFLAMMASOMES	2.06	3.10E-02
REACTOME_DISEASES_OF_CARBOHYDRATE_METABOLISM	2.06	3.10E-02
REACTOME_S_PHASE	2.06	3.12E-02
REACTOME_DNA_REPLICATION	2.06	3.15E-02
REACTOME_DNA_STRAND_ELONGATION	2.06	3.14E-02
WP_ONE_CARBON_METABOLISM	2.05	3.32E-02
REACTOME_CELLULAR_RESPONSE_TO_CHEMICAL_STRESS	2.05	3.31E-02
KEGG_FATTY_ACID_METABOLISM	2.05	3.32E-02
KEGG_TOLL_LIKE_RECEPTOR_SIGNALING_PATHWAY	2.05	3.31E-02
WP_SMALL_CELL_LUNG_CANCER	2.05	3.32E-02
REACTOME_DEX_H_BOX_HELICASES_ACTIVATE_TYPE_I_IFN_AND_INFLAMMATORY_CYTOKINES_PRODUCTION	2.04	3.47E-02
KEGG_BASE_EXCISION_REPAIR	2.03	3.58E-02
REACTOME_ACTIVATED_TAK1_MEDIATES_P38_MAPK_ACTIVATION	2.03	3.59E-02
KEGG_MEDICUS_PATHOGEN_EBV_BARF1_TO_INTRINSIC_APOPTOTIC_PATHWAY	2.03	3.58E-02
WP_MIRNA_ROLE_IN_IMMUNE_RESPONSE_IN_SEPSIS	2.03	3.59E-02
BIOCARTA_CDK5_PATHWAY	2.03	3.63E-02
WP_NANOMATERIAL_INDUCED_APOPTOSIS	2.03	3.64E-02
KEGG_MEDICUS_REFERENCE_CORE_NER_REACTION	2.03	3.66E-02
KEGG_CITRATE_CYCLE_TCA_CYCLE	2.03	3.65E-02
REACTOME_PROTEIN_HYDROXYLATION	2.03	3.63E-02

WP_CILIARY_LANDSCAPE	2.02	3.67E-02
REACTOME_DNA_REPLICATION_PRE_INITIATION	2.02	3.67E-02
REACTOME_P75NTR_SIGNALS_VIA_NF_KB	2.02	3.73E-02
REACTOME_NEF_MEDIATED_DOWNREGULATION_OF_MHC_CLASS_I_COMPLEX_CELL_SURFACE_EXPRESSION	2.02	3.75E-02
BIOCARTA_BIOPEPTIDES_PATHWAY	2.01	3.79E-02
REACTOME_HIV_TRANSCRIPTION_INITIATION	2.01	3.89E-02
WP_AUTOSOMAL_RECESSIVE_OSTEOPETROSIS_PATHWAYS	2.01	3.90E-02
REACTOME_CLASS_I_MHC_MEDIATED_ANTIGEN_PROCESSING_PRESENTATION	2.01	3.96E-02
REACTOME_TRISTETRAPROLIN_TTP_ZFP36_BINDS_AND_DESTABILIZES_MRNA	2.00	3.98E-02
REACTOME_ISG15_ANTIVIRAL_MECHANISM	2.00	4.05E-02
KEGG_MEDICUS_VARIANT_SCRAPIE_CONFORMATION_PRPSC_TO_MGLUR5_CA2_APOPTOTIC_PATHWAY	2.00	4.10E-02
KEGG_MEDICUS_VARIANT_MUTATION_CAUSED_ABERRANT_ABETA_TO_AGE_RAGE_SIGNALING_PATHWAY	2.00	4.15E-02
WP_IL_26_SIGNALING_PATHWAYS	1.99	4.15E-02
REACTOME_FRUCTOSE_METABOLISM	1.99	4.23E-02
WP_BASE_EXCISION_REPAIR	1.99	4.23E-02
KEGG_MAPK_SIGNALING_PATHWAY	1.99	4.28E-02
BIOCARTA_BTG2_PATHWAY	1.99	4.26E-02
BIOCARTA_MAPK_PATHWAY	1.99	4.27E-02
REACTOME_DISEASES_OF_PROGRAMMED_CELL_DEATH	1.98	4.38E-02
WP_FIBRIN_COMPLEMENT_RECEPTOR_3_SIGNALING_PATHWAY	1.98	4.37E-02
REACTOME_CYTOPROTECTION_BY_HMOX1	1.98	4.39E-02
REACTOME_FGFR1_LIGAND_BINDING_AND_ACTIVATION	1.98	4.46E-02
REACTOME_TERMINATION_OF_TRANSLESION_DNA_SYNTHESIS	1.97	4.56E-02
REACTOME_TP53_REGULATES_TRANSCRIPTION_OF_GENES_INVOLVED_IN_CYTOCHROME_C_RELEASE	1.97	4.64E-02
BIOCARTA_MITOCHONDRIA_PATHWAY	1.97	4.62E-02
REACTOME_KETONE_BODY_METABOLISM	1.97	4.62E-02
REACTOME_REGULATION_OF_COMMISSURAL_AXON_PATHFINDING_BY_SLIT_AND_ROBO	1.97	4.61E-02
REACTOME_SIALIC_ACID_METABOLISM	1.97	4.61E-02
KEGG_MEDICUS_VARIANT_MUTATION_INACTIVATED_PINK1_TO_INTRINSIC_APOPTOTIC_PATHWAY_N01050	1.97	4.67E-02
REACTOME_APOPTOSIS	1.97	4.66E-02
HALLMARK_PI3K_AKT_MTOR_SIGNALING	1.96	4.71E-02
REACTOME_G1_S_SPECIFIC_TRANSCRIPTION	1.96	4.70E-02
KEGG_MEDICUS_VARIANT_AMPLIFIED_MDM2_TO_P21_CELL_CYCLE_G1_S	1.96	4.81E-02
WP_ENDOMETRIAL_CANCER	1.96	4.86E-02

HALLMARK_CHOLESTEROL_HOMEOSTASIS	1.95	4.89E-02
PID_IL2_1PATHWAY	1.95	4.93E-02
WP_HOST_PATHOGEN_INTERACTION_OF_HUMAN_CORONAVIRUSES_MAPK_SIGNALING	1.95	4.92E-02
BIOCARTA_P35ALZHEIMERS_PATHWAY	1.95	4.91E-02
KEGG_EPITHELIAL_CELL_SIGNALING_IN_HELICOBACTER_PYLORI_INFECTION	1.95	4.90E-02
REACTOME_PHOSPHOLIPASE_C_MEDIATED_CASCADE_FGFR1	1.95	4.91E-02
REACTOME_BRANCHED_CHAIN_AMINO_ACID_CATABOLISM	1.95	4.90E-02
PID_P73PATHWAY	1.95	4.89E-02
KEGG_MEDICUS_VARIANT_BCL2_OVEREXPRESSION_TO_INTRINSIC_APOPTOTIC_PATHWAY	1.95	4.90E-02
REACTOME_DUAL_INCISION_IN_GG_NER	1.95	4.97E-02
REACTOME_INSULIN_RECEPTOR_RECYCLING	1.94	5.01E-02
WP_UROTENSIN_II_MEDIATED_SIGNALING_PATHWAY	1.94	4.99E-02
HALLMARK_EPITHELIAL_MESENCHYMAL_TRANSITION	-5.59	0.00E+00
REACTOME_EXTRACELLULAR_MATRIX_ORGANIZATION	-5.50	0.00E+00
WP_FOCAL_ADHESION_PI3K_AKT_MTOR_SIGNALING_PATHWAY	-4.02	0.00E+00
REACTOME_INTEGRIN_CELL_SURFACE_INTERACTIONS	-4.01	0.00E+00
HALLMARK_TGF_BETA_SIGNALING	-3.99	0.00E+00
PID_INTEGRIN1_PATHWAY	-3.91	0.00E+00
PID_AVB3_INTEGRIN_PATHWAY	-3.90	0.00E+00
REACTOME_COLLAGEN_FORMATION	-3.78	0.00E+00
KEGG_ECM_RECEPTOR_INTERACTION	-3.70	0.00E+00
REACTOME_ECM_PROTEOGLYCANS	-3.65	0.00E+00
REACTOME_COLLAGEN_DEGRADATION	-3.60	0.00E+00
REACTOME_COLLAGEN_BIOSYNTHESIS_AND_MODIFYING_ENZYMES	-3.54	0.00E+00
WP_PI3K_AKT_SIGNALING_PATHWAY	-3.51	0.00E+00
PID_INTEGRIN3_PATHWAY	-3.51	0.00E+00
REACTOME_ELASTIC_FIBRE_FORMATION	-3.46	0.00E+00
REACTOME_DEGRADATION_OF_THE_EXTRACELLULAR_MATRIX	-3.41	0.00E+00
WP_TGF_BETA_RECEPTOR_SIGNALING_IN_SKELETAL_DYSPLASIAS	-3.41	0.00E+00
WP_PLEURAL_MESOTHELIOMA	-3.39	0.00E+00
KEGG_FOCAL_ADHESION	-3.38	0.00E+00
WP_MIRNA_TARGETS_IN_ECM_AND_MEMBRANE_RECEPTORS	-3.37	0.00E+00
WP_HYPOTHESIZED_PATHWAYS_IN_PATHOGENESIS_OF_CARDIOVASCULAR_DISEASE	-3.29	0.00E+00
REACTOME_SIGNALING_BY_TGFB_FAMILY_MEMBERS	-3.27	0.00E+00
HALLMARK_HYPOXIA	-3.27	0.00E+00
HALLMARK_KRAS_SIGNALING_UP	-3.23	0.00E+00
REACTOME_MOLECULES_ASSOCIATED_WITH_ELASTIC_FIBRES	-3.19	0.00E+00

REACTOME_ASSEMBLY_OF_COLLAGEN_FIBRILS_AND_OTHER_MULTIMERIC_STRUCTURES	-3.18	0.00E+00
KEGG_MEDICUS_REFERENCE_ITGA_B_TALIN_VINCULIN_SIGNALING_PATHWAY	-3.15	0.00E+00
WP_TGF_BETA_RECEPTOR_SIGNALING	-3.14	0.00E+00
WP_A_NETWORK_MAP_OF_MACROPHAGE_STIMULATING_PROTEIN_MSP_SIGNALING	-3.14	0.00E+00
PID_HIF1_TFPATHWAY	-3.08	3.03E-05
WP_NRP1_TRIGGERED_SIGNALING_PATHWAYS_IN_PANCREATIC_CANCER	-3.06	2.94E-05
WP_TYPE_I_COLLAGEN_SYNTHESIS_IN_THE_CONTEXT_OF_OSTEOGENESIS_IMPERFECTA	-3.04	2.84E-05
WP_TGF_SMAD_SIGNALING_PATHWAY	-3.03	5.50E-05
WP_NUCLEAR_RECEPTORS_META_PATHWAY	-3.03	5.34E-05
HALLMARK_UV_RESPONSE_DN	-3.02	5.18E-05
WP_EPITHELIAL_TO_MESENCHYMAL_TRANSITION_IN_COLORECTAL_CANCER	-3.00	5.04E-05
REACTOME_SIGNALING_BY_RECEPTOR_TYROSINE_KINASES	-3.00	7.18E-05
KEGG_MEDICUS_REFERENCE_ITGA_B_RHOGEF_RHOA_SIGNALING_PATHWAY	-2.99	7.00E-05
HALLMARK_GLYCOLYSIS	-2.98	9.08E-05
PID_INTEGRIN5_PATHWAY	-2.98	1.11E-04
WP_FOCAL_ADHESION	-2.91	1.73E-04
KEGG_TGF_BETA_SIGNALING_PATHWAY	-2.91	1.69E-04
PID_UPA_UPAR_PATHWAY	-2.89	1.84E-04
REACTOME_ASPARAGINE_N_LINKED_GLYCOSYLATION	-2.88	1.80E-04
REACTOME_SYNDECAN_INTERACTIONS	-2.87	1.76E-04
REACTOME_COLLAGEN_CHAIN_TRIMERIZATION	-2.85	3.29E-04
REACTOME_ER_TO_GOLGI_ANTEROGRADE_TRANSPORT	-2.84	3.22E-04
KEGG_HYPERTROPHIC_CARDIOMYOPATHY_HCM	-2.82	4.08E-04
REACTOME_NON_INTEGRIN_MEMBRANE_ECM_INTERACTIONS	-2.82	4.54E-04
REACTOME_DISEASES_OF_SIGNAL_TRANSDUCTION_BY_GROWTH_FACTOR_RECEPTORS_AND_SECOND_MESSENGERS	-2.82	4.45E-04
REACTOME_TRANSPORT_TO_THE_GOLGI_AND_SUBSEQUENT_MODIFICATION	-2.80	4.71E-04
WP_CANONICAL_AND_NON_CANONICAL_TGF_B_SIGNALING	-2.79	4.97E-04
REACTOME_REGULATION_OF_INSULIN_LIKE_GROWTH_FACTOR_IGF_TRANSPORT_AND_UPTAKE_BY_INSULIN_LIKE_GROWTH_FACTOR_BINDING_PROTEINS_IGFBPS	-2.79	4.87E-04
KEGG_PATHWAYS_IN_CANCER	-2.78	5.28E-04
REACTOME_SIGNALING_BY_TGF_BETA_RECEPTOR_COMPLEX	-2.77	5.50E-04
KEGG_MEDICUS_PATHOGEN_SHIGELLA_IPAB_C_D_TO_ITGA_B_TALIN_VINCULIN_SIGNALING_PATHWAY	-2.76	5.72E-04

KEGG_CYTOKINE_CYTOKINE_RECEPTOR_INTERACTION	-2.74	6.42E-04
KEGG_MEDICUS_REFERENCE_ITGA_B_FAK_RAC_SIGNALING_PATHWAY	-2.72	7.23E-04
BIOCARTA_CBL_PATHWAY	-2.72	7.56E-04
KEGG_MEDICUS_REFERENCE_ITGA_B_RHOGAP_RHOA_SIGNALING_PATHWAY	-2.71	7.58E-04
REACTOME_SCAVENGING_BY_CLASS_A_RECEPTORS	-2.71	7.45E-04
REACTOME_CIRCADIAN_CLOCK	-2.70	7.62E-04
KEGG_REGULATION_OF_ACTIN_CYTOSKELETON	-2.69	8.07E-04
WP_ANGIOGENESIS	-2.69	8.50E-04
BIOCARTA_ALK_PATHWAY	-2.69	8.37E-04
KEGG_MEDICUS_REFERENCE_ITGA_B_FAK_CDC42_SIGNALING_PATHWAY	-2.67	1.01E-03
WP_INFLAMMATORY_RESPONSE_PATHWAY	-2.64	1.19E-03
WP_EMBRYONIC_STEM_CELL_PLURIPOTENCY_PATHWAYS	-2.62	1.27E-03
KEGG_MEDICUS_REFERENCE_BMP_SIGNALING_PATHWAY	-2.61	1.37E-03
REACTOME_RHO_GTPASE_CYCLE	-2.60	1.52E-03
REACTOME_MYOGENESIS	-2.60	1.56E-03
REACTOME_TGF_BETA_RECEPTOR_SIGNALING_ACTIVATES_SMADS	-2.58	1.86E-03
KEGG_MEDICUS_REFERENCE_HIF_1_SIGNALING_PATHWAY	-2.57	1.85E-03
REACTOME_CROSSLINKING_OF_COLLAGEN_FIBRILS	-2.56	2.04E-03
HALLMARK_MYOGENESIS	-2.56	2.07E-03
REACTOME_MET_ACTIVATES_PTK2_SIGNALING	-2.55	2.14E-03
KEGG_MEDICUS_REFERENCE_PLASMIN_MEDIATED_ACTIVATION_OF_LATENT_TGF_BETA	-2.55	2.18E-03
WP_CLEAR_CELL_RENAL_CELL_CARCINOMA_PATHWAYS	-2.53	2.54E-03
KEGG_MEDICUS_REFERENCE_ITGA_B_RHOG_RAC_SIGNALING_PATHWAY	-2.52	2.61E-03
REACTOME_SIGNALING_BY_NUCLEAR_RECEPTORS	-2.52	2.59E-03
BIOCARTA_TGFB_PATHWAY	-2.52	2.74E-03
WP_ANGIOTENSIN_II_RECEPTOR_TYPE_1_PATHWAY	-2.52	2.71E-03
WP_BURN_WOUND_HEALING	-2.52	2.69E-03
REACTOME_VISUAL_PHOTOTRANSDUCTION	-2.52	2.67E-03
PID_SYNDECAN_1_PATHWAY	-2.51	2.83E-03
REACTOME_SIGNALING_BY_VEGF	-2.48	3.36E-03
PID_FAK_PATHWAY	-2.48	3.36E-03
REACTOME_RUNX2_REGULATES_BONE_DEVELOPMENT	-2.48	3.37E-03
PID_INTEGRIN_CS_PATHWAY	-2.47	3.53E-03
WP_NEOVASCULARISATION_PROCESSES	-2.46	3.88E-03
KEGG_MEDICUS_REFERENCE_TGF_BETA_SIGNALING_PATHWAY	-2.45	4.22E-03
REACTOME_HEMOSTASIS	-2.44	4.30E-03
REACTOME_MET_PROMOTES_CELL_MOTILITY	-2.42	5.01E-03

HALLMARK_MTORC1_SIGNALING	-2.42	5.01E-03
REACTOME_MITOTIC_TELOPHASE_CYTOKINESIS	-2.42	5.05E-03
WP_OSTEOBLAST_SIGNALING	-2.41	5.30E-03
REACTOME_BINDING_AND_UPTAKE_OF_LIGANDS_BY_SCAVENGER_RECEPTORS	-2.40	5.51E-03
WP_HIPPO_MERLIN_SIGNALING_DYSREGULATION	-2.40	5.57E-03
PID_NOTCH_PATHWAY	-2.38	6.15E-03
KEGG_DILATED_CARDIOMYOPATHY	-2.37	6.86E-03
KEGG_ARRHYTHMOGENIC_RIGHT_VENTRICULAR_CARDIOMYOPATHY_ARVC	-2.37	6.81E-03
REACTOME_COHESIN_LOADING_ONTO_CHROMATIN	-2.36	6.90E-03
HALLMARK_APOPTOSIS	-2.35	7.53E-03
PID_VEGFR1_PATHWAY	-2.35	7.85E-03
WP_ARRHYTHMOGENIC_RIGHT_VENTRICULAR_CARDIOMYOPATHY	-2.34	7.88E-03
REACTOME_LAMININ_INTERACTIONS	-2.34	7.82E-03
REACTOME_BACTERIAL_INFECTION_PATHWAYS	-2.34	7.90E-03
WP_TGF_BETA_SIGNALING_PATHWAY	-2.33	8.26E-03
KEGG_MEDICUS_REFERENCE_COHESIN_LOADING	-2.33	8.58E-03
HALLMARK_MITOTIC_SPINDLE	-2.32	8.91E-03
WP_HAIR_FOLLICLE_DEVELOPMENT_ORGANOGENESIS_PART_2_OF_3	-2.31	9.18E-03
REACTOME_NR1H2_AND_NR1H3_MEDIATED_SIGNALING	-2.31	9.29E-03
WP_PLATELET_MEDIATED_INTERACTIONS_WITH_VASCULAR_AND_CIRCULATING_CELLS	-2.31	9.43E-03
REACTOME_RUNX2_REGULATES_OSTEOBLAST_DIFFERENTIATION	-2.30	9.51E-03
PID_INTEGRIN_A9B1_PATHWAY	-2.30	9.56E-03
REACTOME_SIGNALING_BY_BMP	-2.30	9.56E-03
REACTOME_DEADENYLATION_OF_MRNA	-2.30	9.55E-03
REACTOME_EPHB_MEDIATED_FORWARD_SIGNALING	-2.30	9.71E-03
BIOCARTA_HIF_PATHWAY	-2.29	9.81E-03
WP_TRANSLATION_INHIBITORS_IN_CHRONICALLY_ACTIVATED_PDGFRA_CELLS	-2.29	9.81E-03
REACTOME_DOWNREGULATION_OF_SMAD2_3_SMAD4_TRANSCRIPTIONAL_ACTIVITY	-2.29	9.79E-03
PID_VEGF_VEGFR_PATHWAY	-2.29	9.95E-03
KEGG_CIRCADIAN_RHYTHM_MAMMAL	-2.28	1.02E-02
HALLMARK_COAGULATION	-2.27	1.08E-02
REACTOME_RESPONSE_TO_ELEVATED_PLATELET_CYTOSOLIC_CA2	-2.27	1.08E-02
REACTOME_SIGNALING_BY_PDGF	-2.27	1.12E-02
PID_ALK1_PATHWAY	-2.25	1.28E-02
HALLMARK_PROTEIN_SECRETION	-2.24	1.29E-02
BIOCARTA_LYMPHOCYTE_PATHWAY	-2.24	1.29E-02

REACTOME_UNFOLDED_PROTEIN_RESPONSE_UPR	-2.24	1.33E-02
WP_CELL_INTERACTIONS_OF_THE_PANCREATIC_CANCER_MICROENVIRONMENT	-2.23	1.36E-02
BIOCARTA_MONOCYTE_PATHWAY	-2.23	1.38E-02
WP_GLYCOSAMINOGLYCAN_SYNTHESIS_IN_FIBROBLASTS	-2.23	1.39E-02
REACTOME_UPTAKE_AND_ACTIONS_OF_BACTERIAL_TOXINS	-2.21	1.52E-02
PID_BETA_CATENIN_NUC_PATHWAY	-2.21	1.55E-02
PID_BMP_PATHWAY	-2.20	1.62E-02
WP_HFE_EFFECT_ON_HEPCIDIN_PRODUCTION	-2.19	1.71E-02
REACTOME_PLATELET_ACTIVATION_SIGNALING_AND_AGGREGATION	-2.19	1.70E-02
REACTOME_THE_CANONICAL_RETINOID_CYCLE_IN_RODS_TWILIGHT_VISION	-2.19	1.72E-02
WP_METABOLIC_PATHWAYS_OF_FIBROBLASTS	-2.19	1.74E-02
WP_TGF_BETA_SIGNALING_IN_THYROID_CELLS_FOR_EPITHELIAL_MESENCHYMAL_TRANSITION	-2.18	1.83E-02
PID_LYMPH_ANGIOGENESIS_PATHWAY	-2.17	1.93E-02
REACTOME_GASTRIN_CREB_SIGNALLING_PATHWAY_VIA_PKC_AND_MAPK	-2.17	1.92E-02
HALLMARK_ANDROGEN_RESPONSE	-2.17	1.91E-02
REACTOME_NOTCH2_INTRACELLULAR_DOMAIN_REGULATES_TRANSCRIPTION	-2.17	1.92E-02
REACTOME_INTRA_GOLGI_AND_RETROGRADE_GOLGI_TO_ER_TRAFFIC	-2.17	1.93E-02
REACTOME_ONCOGENIC_MAPK_SIGNALING	-2.17	1.92E-02
KEGG_MEDICUS_PATHOGEN_HPV_E6_TO_NOTCH_SIGNALING_PATHWAY_N00381	-2.16	1.97E-02
REACTOME_LOSS_OF_FUNCTION_OF_SMAD2_3_IN_CANCER	-2.16	2.00E-02
PID_SYNDECAN_4_PATHWAY	-2.15	2.03E-02
WP_NANOPARTICLE_MEDIATED_ACTIVATION_OF_RECEPTOR_SIGNALING	-2.15	2.06E-02
KEGG_LEUKOCYTE_TRANSENDOTHELIAL_MIGRATION	-2.15	2.11E-02
REACTOME_RND2_GTPASE_CYCLE	-2.13	2.33E-02
REACTOME_COPI_MEDIATED_ANTEROGRADE_TRANSPORT	-2.13	2.32E-02
KEGG_MTOR_SIGNALING_PATHWAY	-2.13	2.34E-02
WP_MFAP5_MEDIATED_OVARIAN_CANCER_CELL_MOTILITY_AND_INVASIVENESS	-2.13	2.34E-02
REACTOME_N_GLYCAN_TRIMMING_IN_THE_ER_AND_CALNEXIN_CALRETICULIN_CYCLE	-2.12	2.37E-02
KEGG_MEDICUS_REFERENCE_WEE1_CELL_CYCLE_G2_M	-2.12	2.38E-02
WP_IL_24_SIGNALING_PATHWAY	-2.10	2.69E-02
WP_HAIR_FOLLICLE_DEVELOPMENT_CYTODIFFERENTIATION_PART_3_OF_3	-2.10	2.69E-02

KEGG_MEDICUS_REFERENCE_AMH_SIGNALING_PATHWAY	-2.10	2.80E-02
REACTOME_REGULATION_OF_CDH11_FUNCTION	-2.08	3.03E-02
PID_INTEGRIN_A4B1_PATHWAY	-2.08	3.10E-02
WP_OSTEOBLAST_DIFFERENTIATION_AND_RELATED_DISEASES	-2.08	3.13E-02
HALLMARK_ANGIOGENESIS	-2.08	3.16E-02
KEGG_FC_GAMMA_R_MEDIATED_PHAGOCYTOSIS	-2.07	3.18E-02
WP_DIFFERENTIATION_OF_WHITE_AND_BROWN_ADIPOCYTE	-2.07	3.16E-02
WP_EXTRACELLULAR_VESICLE_MEDIATED_SIGNALING_IN_RECIPIENT_CELLS	-2.07	3.19E-02
WP_WNT_SIGNALING	-2.06	3.38E-02
WP_REGULATORY_CIRCUITS_OF_THE_STAT3_SIGNALING_PATHWAY	-2.06	3.42E-02
WP_MICRORNA_FOR_TARGETING_CANCER_GROWTH_AND_VASCULARIZATION_IN_GLIOBLASTOMA	-2.06	3.43E-02
REACTOME_REGULATION_OF_HOMOTYPIC_CELL_CELL_ADHESION	-2.05	3.43E-02
REACTOME_AURKA_ACTIVATION_BY_TPX2	-2.05	3.42E-02
KEGG_ASTHMA	-2.05	3.43E-02
WP_ROLE_OF_HYPOXIA_ANGIOGENESIS_AND_FGF_PATHWAY_IN_OA_CHONDROCYTE_HYPERTROPHY	-2.05	3.42E-02
WP_CLOCK_CONTROLLED_AUTOPHAGY_IN_BONE_METABOLISM	-2.05	3.43E-02
WP_GLUCOCORTICOID_RECEPTOR_PATHWAY	-2.05	3.43E-02
KEGG_MEDICUS_REFERENCE_BMP_HAMP_SIGNALING_PATHWAY	-2.04	3.58E-02
REACTOME_RND3_GTPASE_CYCLE	-2.04	3.64E-02
REACTOME_TRANSCRIPTIONAL_ACTIVITY_OF_SMAD2_SMAD3_SMAD4_HETEROTRIMER	-2.04	3.62E-02
KEGG_MEDICUS_REFERENCE_REGULATION_OF_GF_RTK_RAS_ERK_SIGNALING_UBIQUITINATION_OF_RTK_BY_CBL	-2.03	3.79E-02
REACTOME_SIGNALING_BY_MET	-2.03	3.89E-02
REACTOME_ACTIVATION_OF_MATRIX_METALLOPROTEINASES	-2.02	3.92E-02
REACTOME_SPECIFICATION_OF_PRIMORDIAL_GERM_CELLS	-2.02	3.91E-02
REACTOME_PRE_NOTCH_PROCESSING_IN_GOLGI	-2.02	3.92E-02
REACTOME_NFE2L2_REGULATING_ANTI_OXIDANT_DETOXIFICATION_ENZYMES	-2.02	4.00E-02
WP_ENDOCHONDRAL_OSSIFICATION	-2.01	4.07E-02
REACTOME_RHO_GTPASE_EFFECTORS	-2.01	4.05E-02
WP_OVERLAP_BETWEEN_SIGNAL_TRANSDUCTION_PATHWAYS_CONTRIBUTING_TO_LMNA_LAMINOPATHIES	-2.01	4.07E-02
WP_NIPBL_ROLE_IN_DNA_DAMAGE_CORNELIA_DE_LANGE_SYNDROME	-2.01	4.10E-02
REACTOME_SERINE_BIOSYNTHESIS	-2.01	4.11E-02
WP_GENES_CONTROLLING_NEPHROGENESIS	-2.00	4.23E-02
PID_P38_GAMMA_DELTA_PATHWAY	-2.00	4.22E-02
REACTOME_COPII_MEDIATED_VESICLE_TRANSPORT	-2.00	4.21E-02

KEGG_MEDICUS_REFERENCE_EGF_EGFR_ACTIN_SIGNALING_PATHWAY	-2.00	4.22E-02
PID_CDC42_PATHWAY	-2.00	4.22E-02
WP_ADIPOGENESIS	-2.00	4.34E-02
WP_PHOTODYNAMIC_THERAPY_INDUCED_AP_1_SURVIVAL_SIGNALING	-1.99	4.34E-02
REACTOME_CELL_JUNCTION_ORGANIZATION	-1.99	4.40E-02
PID_ALK2_PATHWAY	-1.99	4.46E-02
WP_TH17_CELL_DIFFERENTIATION_PATHWAY	-1.99	4.47E-02
WP_ENDOCHONDRAL_OSSIFICATION_WITH_SKELETAL_DYSPLASIAS	-1.99	4.46E-02
WP_MESODERMAL_COMMITMENT_PATHWAY	-1.98	4.50E-02
REACTOME_CHONDROITIN_SULFATE_DERMATAN_SULFATE_METABOLISM	-1.98	4.49E-02
PID_EPHRINB_REV_PATHWAY	-1.98	4.58E-02
REACTOME_RETINOID_CYCLE_DISEASE_EVENTS	-1.98	4.58E-02
REACTOME_CALNEXIN_CALRETICULIN_CYCLE	-1.98	4.64E-02
WP_ANDROGEN_RECEPTOR_SIGNALING_PATHWAY	-1.97	4.73E-02
WP_CKAP4_SIGNALING_PATHWAY_MAP	-1.97	4.77E-02
WP_COMPLEMENT_SYSTEM	-1.97	4.84E-02
REACTOME_SIGNALING_BY_TGF_BETA_RECEPTOR_COMPLEX_IN_CANCER	-1.97	4.84E-02
REACTOME_CELL_SURFACE_INTERACTIONS_AT_THE_VASCULAR_WALL	-1.96	4.88E-02

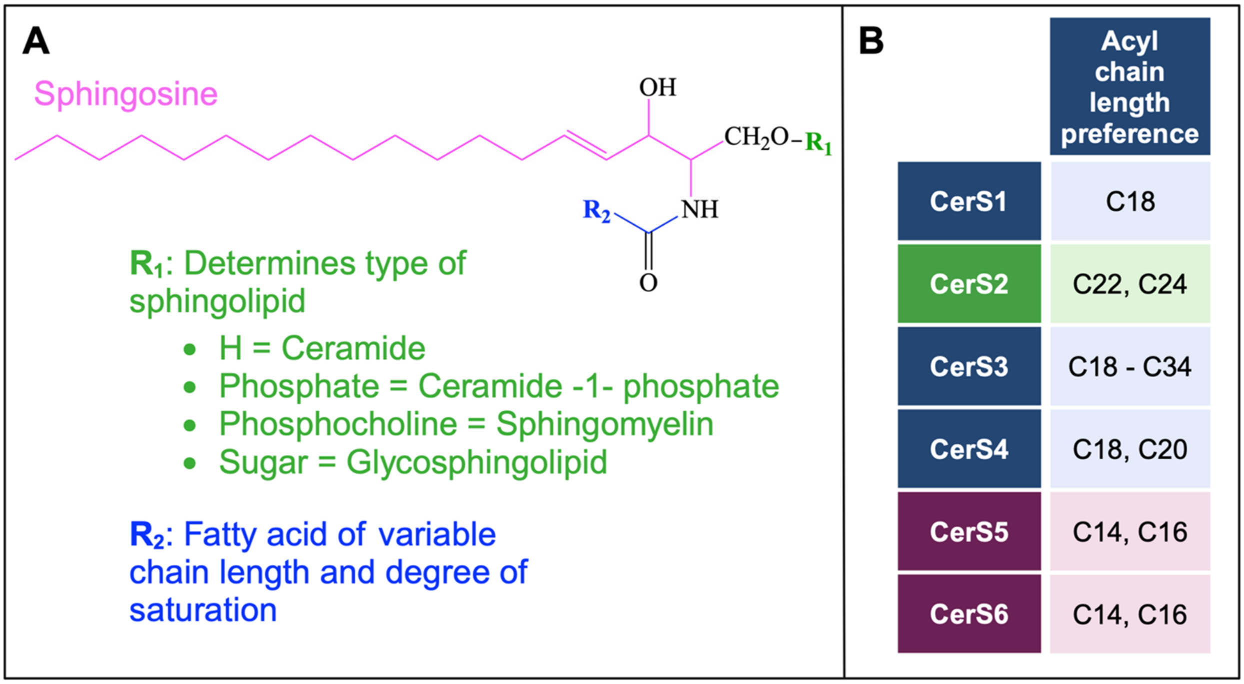

Supplementary Figure S1. Acyl chain length preference of the mammalian CerS enzymes. (**A**) The general components of a sphingolipid with a sphingosine backbone, varying fatty acid chain lengths and degrees of saturation acylated to this backbone (R_2), and a head group (R_1) determining the relevant sphingolipid class. (**B**) The different ceramide synthases (CerS) and their preferences in acyl chain length resulting in the generation of the corresponding ceramide. CerS2 is selective for the production of very long-chain ceramides (22:0 and 24:0) while CerS5 and CerS6 generate long chain ceramide (16:0). Created with BioRender.com

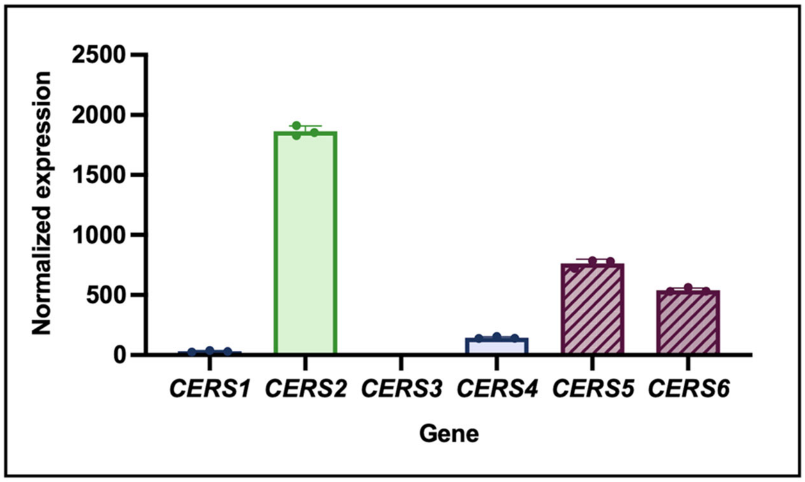

Supplementary Figure S2. CERS gene expression in HCMs from RNA-sequencing data. Normalized CERS expression confirming the expression of the different *CERS* in immortalized human ventricular cardiomyocytes, n=3.

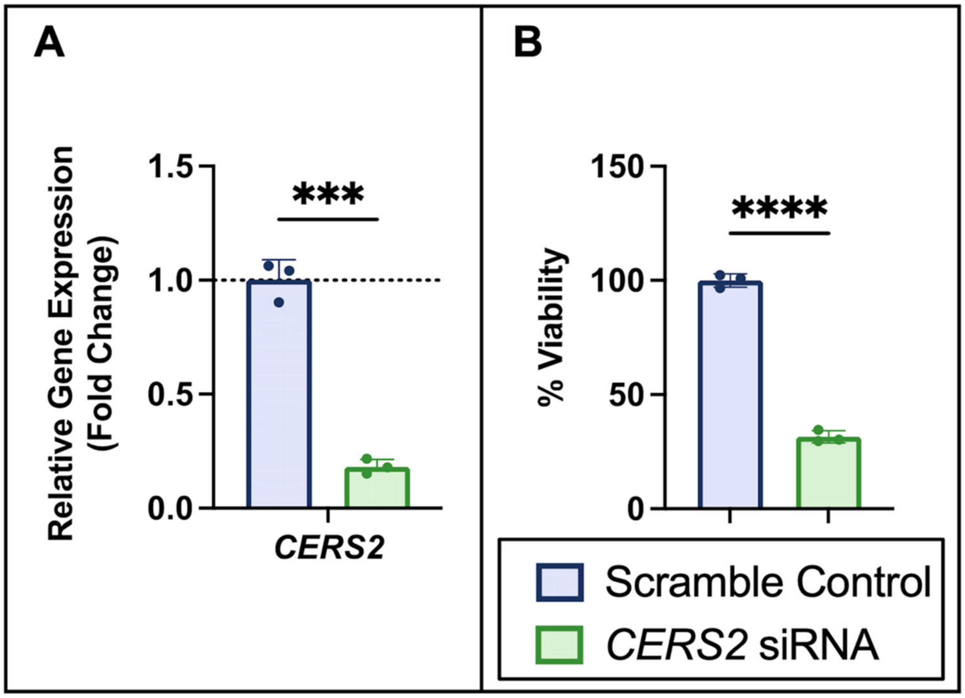

Supplementary Figure S3. Optimized ~ 80% *CERS2* KD leads to significant cell death. (**A**) Treatment with *CERS2* siRNA leads to 82% KD of *CERS2*. (**B**) Cell viability following 72h knockdown of *CERS2* compared to the scramble control. n=3, **** P <0.0001, *** P < 0.001

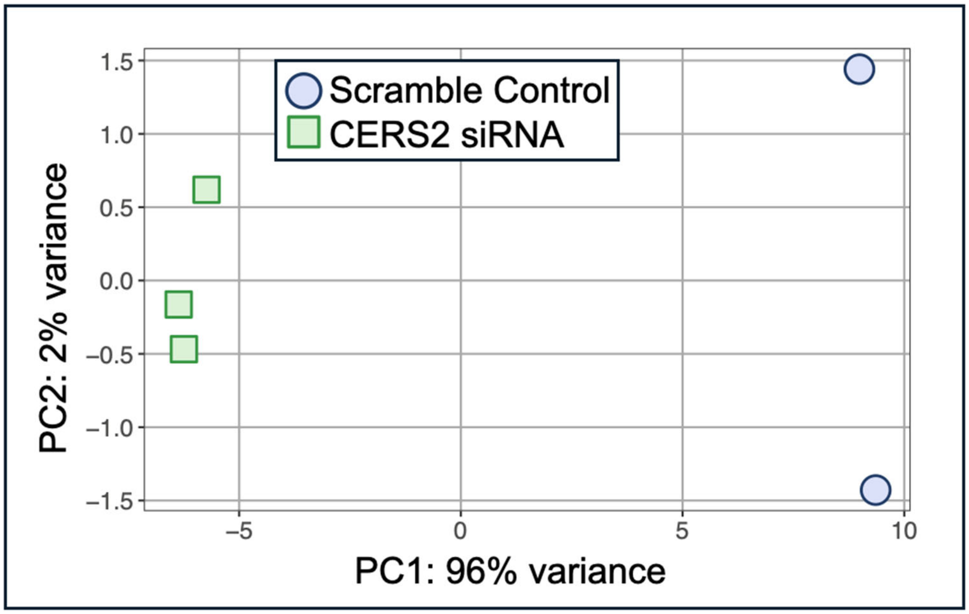

Supplementary Figure S4. Transcriptome variation between *CERS*2 KD and scramble control samples. PCA plot across the HCM transcriptome in *CERS2* KD samples (green boxes) and scramble control (blue circles).

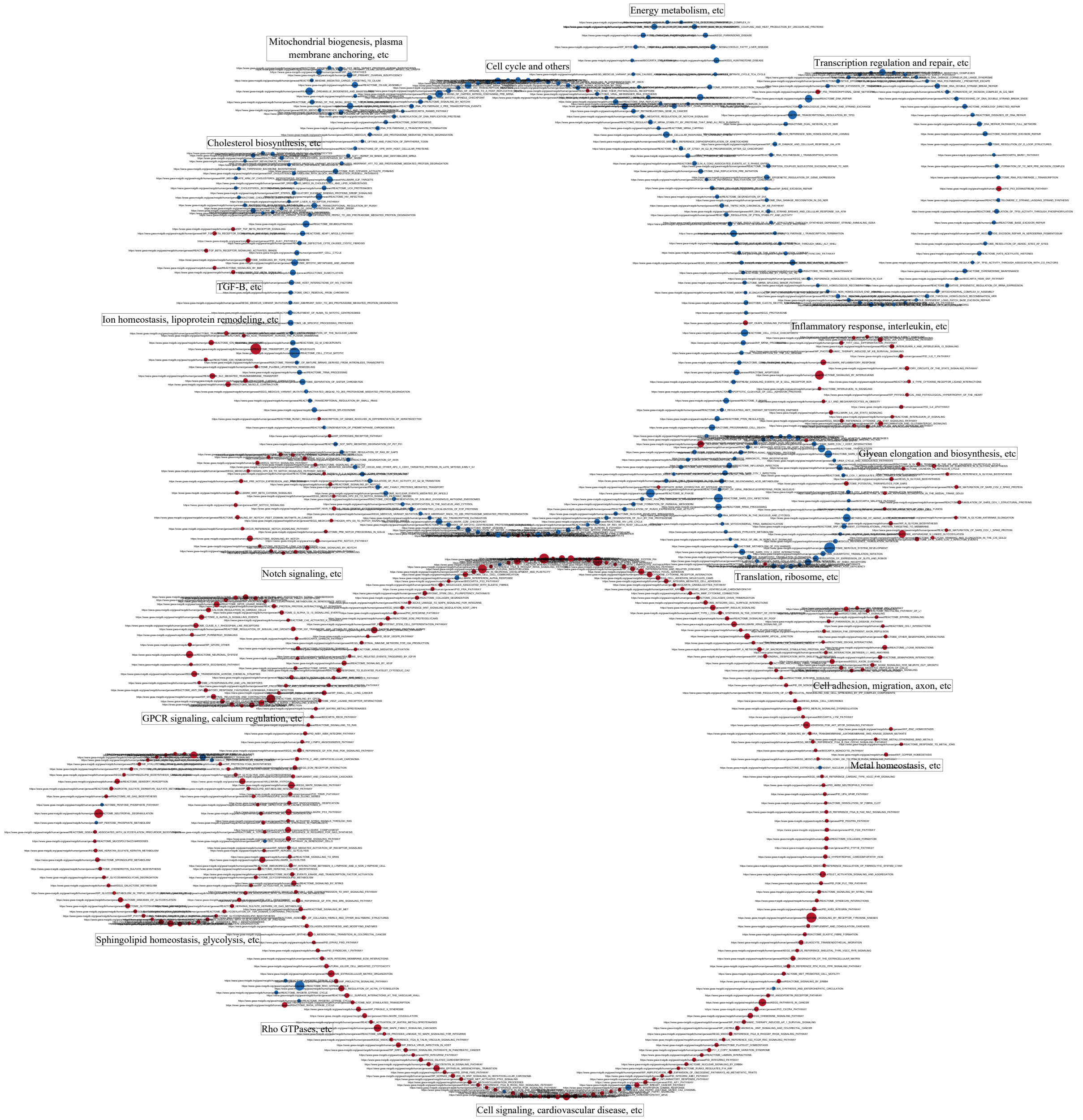

Supplementary Figure S5. Network based visual depiction of GSEA results after *CERS2* knockdown in human ventricular cardiomyocytes. Red spheres and blue spheres correspond to upregulated and downregulated gene sets, respectively. Connectivity between the gene sets demonstrates greater than 50% overlap among member genes.

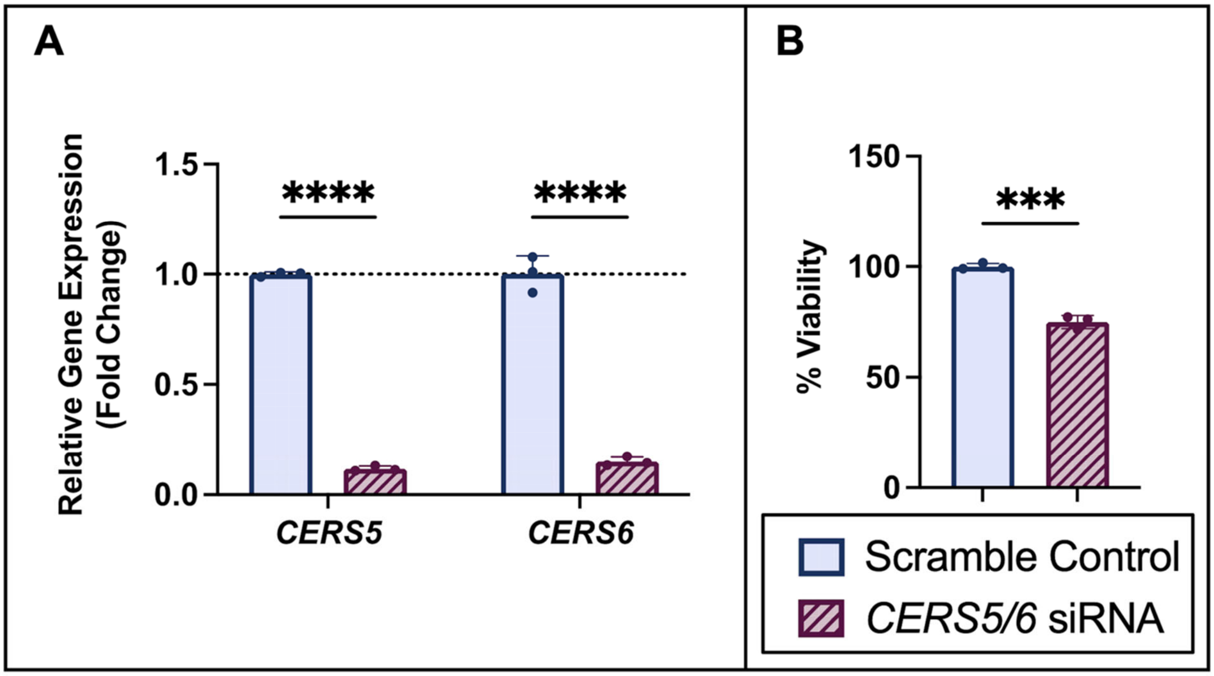

Supplementary Figure S6. Optimized ~80% *CERS5/6* KD leads to small amounts of cell death. (**A**) Treatment with *CERS5* and *CERS6* siRNA leads to 88% KD of *CERS5* and 85% KD of *CERS6*. (**B**) HCM viability declines to 75% following 72h *CERS5* and *CERS6* KD. n=3, **** $P < 0.0001$, *** $P < 0.001$

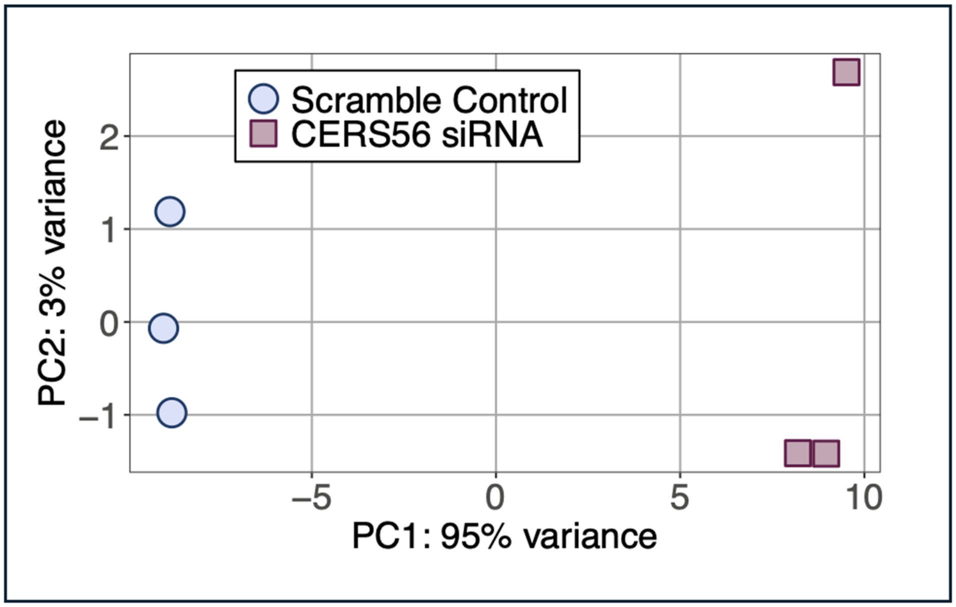

Supplementary Figure S7. Transcriptome variation between *CERS*5/6 KD and scramble control samples. PCA plot across the HCM transcriptome in *CERS5/6* KD samples (purple boxes) and scramble control (blue circles).

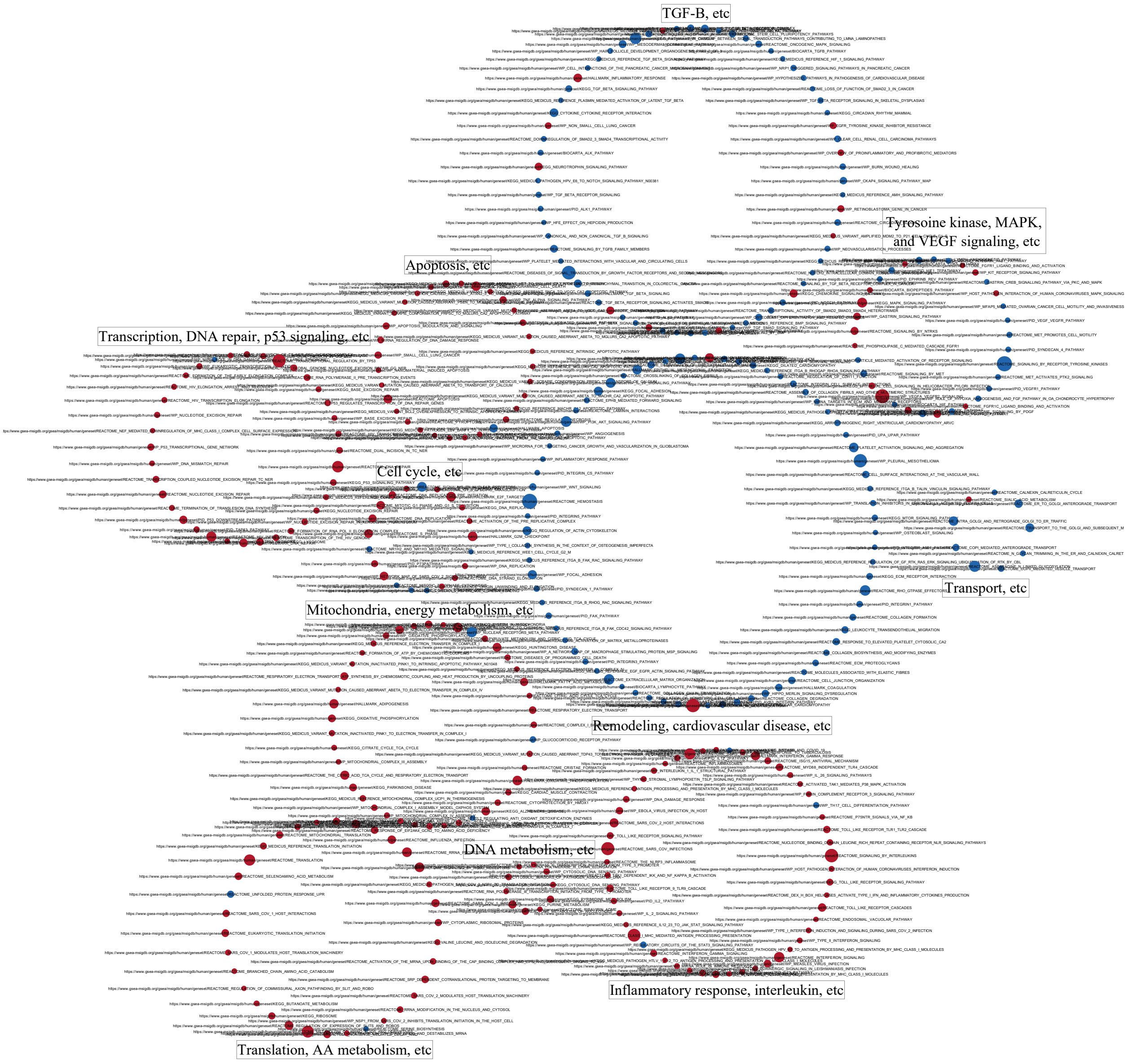

Supplementary Figure S8. Network based visual depiction of GSEA results after *CERS5/6* knockdown in human ventricular cardiomyocytes. Red spheres and blue spheres correspond to upregulated and downregulated gene sets, respectively, while connectivity between the gene sets demonstrates greater than 50% overlap among member genes.
